# Supplementary material for: Plasmon–emitter interactions at the nanoscale
Source: Nat Commun. 2020 Jan 17;11:366. doi: 10.1038/s41467-019-13820-z (PMC6969188; doi:10.1038/s41467-019-13820-z)
Supplement: Supplementary file 2 — Supplementary Information [file 41467_2019_13820_MOESM2_ESM.pdf]

SUPPLEMENTARY INFORMATION
Plasmon–Emitter Interactions at the Nanoscale

P. A. D. Gonçalves,[1,2,3,4,*] Thomas Christensen,[1,†] Nicholas Rivera,[1] Antti-Pekka Jauho,[3,5]
N. Asger Mortensen,[2,3,6,‡] and Marin Soljačić[1]
[1]*Department of Physics, Massachusetts Institute of Technology, Cambridge, MA 02139, USA*
[2]*Center for Nano Optics, University of Southern Denmark, Campusvej 55, DK-5230 Odense M, Denmark*
[3]*Center for Nanostructured Graphene, Technical University of Denmark, DK-2800 Kgs. Lyngby, Denmark*
[4]*Department of Photonics Engineering, Technical University of Denmark, DK-2800 Kgs. Lyngby, Denmark*
[5]*Department of Physics, Technical University of Denmark, DK-2800 Kgs. Lyngby, Denmark*
[6]*Danish Institute for Advanced Study, University of Southern Denmark, Campusvej 55, DK-5230 Odense M, Denmark*

SUPPLEMENTARY NOTE 1. FEIBELMAN *d*-PARAMETERS

Classical descriptions of photonic and plasmonic systems rely on the knowledge of the materials' local bulk dielectric functions (or conductivities) [1; 2]. These, together with the specification of the structure's geometry (e.g., size and shape) constitute the sole ingredients required for computing the electromagnetic response of the system and collective excitations [1–3]. Notwithstanding, as the characteristic dimension of metallic nanostructures reaches the few-nanometer regime, the accurateness of traditional descriptions in terms of classical electrodynamics rapidly declines owing to the emergence of nonlocal and quantum mechanical effects [3–6]. In general, the exact computation of the optical response of a quantum nanoplasmonic system is a challenging task due to the complex nonlocal dynamics of the plasmon-supporting electron gas. One approach relies on the use of *ab initio* time-dependent density functional theory (TDDFT) [7] in an attempt to describe systems' response in a quantum mechanical framework (where the effects included naturally depend on the approximation(s) employed when solving the many-body problem). Nonetheless, techniques fully based on TDDFT are in practice limited to very small systems like metal clusters, whose radii typically falls below $\lesssim 3$ nm (for instance, a Na cluster with around 1000 atoms would span a radius of only about 2 nm [8]). However, the vast majority of nanoplasmonic structures of interest possess characteristic length scales that can be significantly larger than that, though not large enough to be described in terms of classical electrodynamics. Hence, it becomes clear that there is a high-demand for the development of suitable theoretical methods for quantum nanoplasmonics that are capable to treat quantum and nonlocal effects rigorously, while at the same time being relatively simple to implement. This is the approach we pursue here. Specifically, we extend and further develop the formalism of Feibelman d-parameters [4; 9] in order to bridge the "classical to quantum boundary" (cf. Figure 1 of the main text). This framework is rooted on the introduction of quantum surface-response functions—here obtained using TDDFT—dubbed as Feibelman $d_\perp$- and $d_\parallel$-parameters. These account for the dynamics of the surface region where the electron gas is inhomogeneous, whereas the bulk is still described in terms of classical (local) response functions—see Supplementary Figure 1. For an introduction to this topic, we refer to Refs. 3, 4, and 9.

The formulation in terms of the d-parameters can therefore take into account, to leading-order, intrinsically quantum mechanical effects such as electronic spill-out (or "spill-in"), nonlocality, and surface-enabled Landau damping (i.e., plasmon decay into electron-hole pairs), while preserving an amenable theoretical treatment that is general and that can be practically implemented in a plethora of plasmonic nanostructures [3; 10].

The Feibelman d-parameters can be defined via the quantum mechanical induced charge density, $\rho(\mathbf{r}) \equiv \rho(z)\mathrm{e}^{\mathrm{i}qx}$, and corresponding induced current density, $\mathbf{J}(\mathbf{r}) \equiv \mathbf{J}(z)\mathrm{e}^{\mathrm{i}qx}$, via[1] [3; 4; 9]

$$d_\perp(\omega) = \frac{\int_{-\infty}^{\infty} z\rho(z)\,\mathrm{d}z}{\int_{-\infty}^{\infty} \rho(z)\,\mathrm{d}z} \qquad \text{and} \qquad d_\parallel(\omega) = \frac{\int_{-\infty}^{\infty} z\,\partial_z J_x(z)\,\mathrm{d}z}{\int_{-\infty}^{\infty} \partial_z J_x(z)\,\mathrm{d}z}. \tag{1}$$

[1] In general, the d-parameters depend both on frequency and momentum. The latter dependence, however, is relatively weak [10], and therefore negligible to leading order; thus, we concern ourselves only with their frequency-dependence.

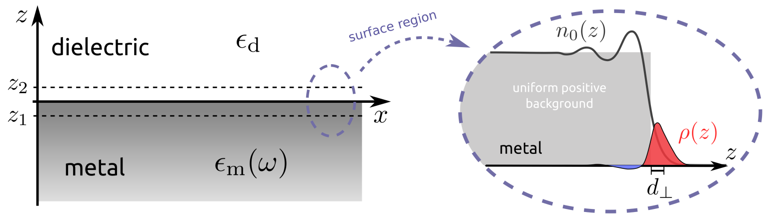

Supplementary Figure 1. Illustration of a planar dielectric–metal interface, defined by the $z = 0$ plane. In the asymptotic regions, i.e., for $|z| \geq |z_{1,2}|$, the cladding dielectric is characterized by a dielectric constant, ϵ_d, whereas the electromagnetic properties of the metal are accounted for by its (local) bulk dielectric function, $\epsilon_m(\omega)$. In the surface region, quantum mechanical features lead to a nonuniform equilibrium electron density, $n_0(z)$, and an induced charge density, $\rho(z)$ (that arises due to the system's response to an external perturbation). As indicated schematically in the figure, the Feibelman $d_\perp$ parameter can be regarded as the position of the centroid of the induced charge density with respect to the positive background edge.

where it is implicit that the interface runs along the x-direction and the z-direction lies normal to it, as depicted in Supplementary Figure 1. It is apparent from Eqs. (1) that $d_\perp$ is a length scale defined by the position of the centroid of the induced charge density (see Supplementary Figure 1) whereas $d_\parallel$ can be regarded as the (integrated) imbalance between the uniform ionic background and n_0 [3]. Hence, the latter (to leading-order) is identically zero for charge-neutral interfaces [4]. Once the microscopic variations of the quantum mechanical charge and current densities across the dielectric–metal boundary (here defined by the so-called jellium edge) are computed, e.g., using TDDFT, one can derive the corresponding nonclassical reflection and transmission coefficients. These quantities entail all the knowledge about the system's near- and far-field electromagnetic response; therefore, they provide us with, for instance, the structure's plasmon resonances, Purcell enhancement, and other nanophotonic phenomena.

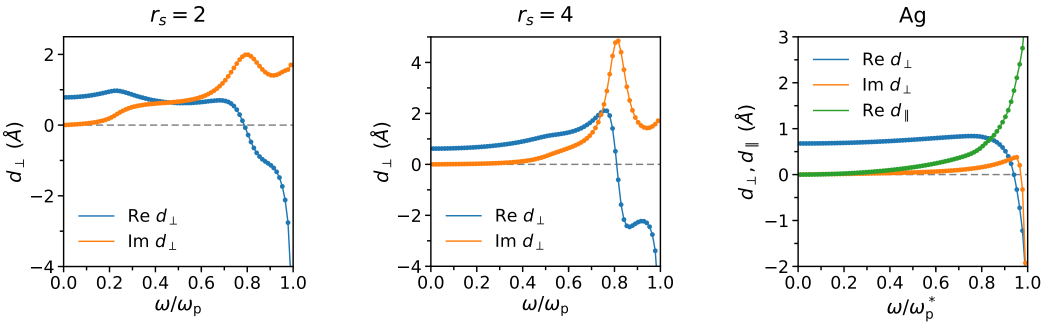

Supplementary Figure 2. Spectral dependence of the Feibelman d-parameters for jellium metals with different Wigner-Seitz radius, $r_s = \{2, 4\}$ (in units of the Bohr radius), and for silver (Ag). We note that a jellium with $r_s = \{2, 4\}$ is resemblant of aluminum (Al) and sodium (Na), respectively. For silver (Ag), we normalize the frequencies to the *screened* plasma frequency, $\omega_p^* \simeq 3.81$ eV. The circles represent data obtained from TDDFT calculations by Christensen *et al.* [3] and the solid lines are the corresponding interpolations. The dielectric medium interfacing the metal is assumed to have $\epsilon_d = 1$.

Figure 2 shows the Feibelman d-parameters obtained previously in Ref. 3 through TDDFT calculations within the jellium model for the interacting electron gas. The figure depicts the d-parameters for simple metals with different electronic densities, defined by the Wigner–Seitz radius $r_s = \{2, 4\}$ (in units of the Bohr radius, a_B), via $r_s a_B = (3/4\pi n)^{1/3}$. The corresponding plasma frequency is thus $\omega_p = \sqrt{\frac{3e^2}{4\pi\epsilon_0 m}\frac{1}{(r_s a_B)^3}}$. It should be noted that the d-parameters are microscopic surface-response functions that obey Kramers–Kronig relations and specific sum-rules [11], just like the traditional bulk response functions do [12]. The TDDFT data presented here is for air–metal (or vacuum–metal) interfaces (i.e., for $\epsilon_d = 1$); we note that the specific values of the d-parameters depend on the dielectric medium next to the metal [4; 13], since it changes the screened interaction.

Lastly, we emphasize that although in Supplementary Figure 2 we have $d_\parallel \neq 0$ for silver (last panel in Supplementary Figure 2) in this case silver is of course still charge-neutral. The finiteness of $d_\parallel$ here is

merely a consequence of the methodology used to incorporate d-band screening in the TDDFT calculation [3] (see Refs. 3 and 4 for additional details).

SUPPLEMENTARY NOTE 2. MODIFIED BOUNDARY CONDITIONS WITH FEIBELMAN *d*-PARAMETER SURFACE TERMS

Here we summarize the recently introduced approach to incorporate the Feibelman d-parameters as boundary conditions applied to the conventional macroscopic Maxwell equations [14]. The boundary conditions facilitate a convenient and straightforward d-parameter generalization of conventional scattering coefficients—e.g., the reflection and transmission coefficients of a planar interface, or the Mie coefficients of a spherical interface—thereby providing the necessary ingredients to investigate the influence of quantum mechanical effects on the surface plasmon polariton (SPP) dispersion, electromagnetic local density of states (LDOS), etc.

In the absence of external charges and currents, the classical boundary conditions of macroscopic electrodynamics at an interface separating two media read [15]:

$$\hat{\mathbf{n}} \times (\mathbf{E}_2 - \mathbf{E}_1) = 0, \tag{2a}$$
$$\hat{\mathbf{n}} \times (\mathbf{H}_2 - \mathbf{H}_1) = 0, \tag{2b}$$

where $\hat{\mathbf{n}}$ is a unit vector normal to the interface $\partial\Omega$ (pointing from medium 1 to medium 2). These govern the tangential (or parallel) components of the electromagnetic fields, imposing their continuity across the interface. However, as can be clearly seen in a nonretarded formulation [3; 4], the introduction of the Feibelman d-parameters renders the above boundary conditions inapplicable. Hence, a revision set of boundary conditions which reflect the presence of the d-parameters is required. The incorporation can be inferred by considering their impact as initially encoded through (normally-oriented) surface polarization and (tangentially-oriented) current terms, $\mathbf{P}(\mathbf{r}) = \boldsymbol{\pi}(\mathbf{r})\delta(\mathbf{r} - \mathbf{r}_{\partial\Omega})$ and $\mathbf{J}(\mathbf{r}) = \mathbf{K}(\mathbf{r})\delta(\mathbf{r} - \mathbf{r}_{\partial\Omega})$, respectively, which are nonzero only at the interface, i.e., at $\mathbf{r} = \mathbf{r}_{\partial\Omega}$. The surface polarization and current densities are each proportional to a d-parameter and are driven by the discontinuities of the field components [14]

$$\boldsymbol{\pi} \equiv \epsilon_0 d_\perp \left[\hat{\mathbf{n}} \cdot (\mathbf{E}_2 - \mathbf{E}_1)\right] \hat{\mathbf{n}}, \tag{3a}$$
$$\mathbf{K} \equiv \mathrm{i}\omega d_\parallel \left[\hat{\mathbf{n}} \times (\mathbf{D}_2 - \mathbf{D}_1) \times \hat{\mathbf{n}}\right]. \tag{3b}$$

After some manipulations, these surface polarizations and currents can be self-consistently absorbed into a revision of the conventional boundary conditions[2] [14]

$$\begin{aligned} \hat{\mathbf{n}} \times (\mathbf{E}_2 - \mathbf{E}_1) &= \hat{\mathbf{n}} \times \left[-\epsilon_0^{-1} \nabla_\parallel (\boldsymbol{\pi} \cdot \hat{\mathbf{n}})\right] \\ &= -d_\perp \hat{\mathbf{n}} \times \left[\nabla_\parallel \hat{\mathbf{n}} \cdot (\mathbf{E}_2 - \mathbf{E}_1)\right], \end{aligned} \tag{4a}$$
$$\hat{\mathbf{n}} \times (\mathbf{H}_2 - \mathbf{H}_1) = \mathrm{i}\omega d_\parallel \left[\hat{\mathbf{n}} \times (\mathbf{D}_2 - \mathbf{D}_1) \times \hat{\mathbf{n}}\right], \tag{4b}$$

where $\nabla_\parallel$ is the surface nabla operator $\nabla_\parallel \equiv (1 - \hat{\mathbf{n}}\hat{\mathbf{n}}^{\mathrm{T}})\nabla$. The boundary conditions can be equivalently stated via the parallel and perpendicular components (with respect to the interface) of the fields as

$$\mathbf{E}_{2,\parallel} - \mathbf{E}_{1,\parallel} = -d_\perp \nabla_\parallel (E_{2,\perp} - E_{1,\perp}), \tag{5a}$$
$$\mathbf{H}_{2,\parallel} - \mathbf{H}_{1,\parallel} = \mathrm{i}\omega d_\parallel (\mathbf{D}_{2,\parallel} - \mathbf{D}_{1,\parallel}) \times \hat{\mathbf{n}}. \tag{5b}$$

Clearly, the incorporation of Feibelman d-parameters introduces discontinuities in the parallel components of the electric and magnetic fields. The magnitude of these discontinuities is naturally proportional to the Feibelman d-parameters.

---

[2] While the boundary conditions on the tangential parts of **E** and **H** is fully sufficient to uniquely couple solutions across the interface, the complementary set of boundary conditions—on the normal components of **B** and **D**—is occasionally more convenient. The boundary condition for the normal components of **B**, is unchanged from its classical counterpart, i.e., $\hat{\mathbf{n}} \cdot (\mathbf{B}_2 - \mathbf{B}_1) = 0$; for the normal components of **D**, it is $\hat{\mathbf{n}} \cdot (\mathbf{D}_2 - \mathbf{D}_1) = d_\parallel \nabla_\parallel \cdot [\hat{\mathbf{n}} \times (\mathbf{D}_2 - \mathbf{D}_1) \times \hat{\mathbf{n}}]$.

SUPPLEMENTARY NOTE 3. NONCLASSICAL REFLECTION AND TRANSMISSION COEFFICIENTS FOR A PLANAR DIELECTRIC–METAL INTERFACE

In possession of the boundary conditions personified by Eqs. (5), we now have all the necessary ingredients to determine the nonclassical equivalents of Fresnel's reflection and transmission coefficients for a single dielectric–metal interface. In what follows, we adopt the same coordinate system as in Supplementary Figure 1.

Nonclassical reflection and transmission coefficients for TM waves (*p*-polarization)

We seek transverse magnetic (TM) solutions, in the dielectric and metal half-spaces, of the form:

$$\mathbf{H}_\mathrm{d} = \left(\mathrm{e}^{-\mathrm{i}k_{z,\mathrm{d}}z} + r_p^{(H)}\mathrm{e}^{\mathrm{i}k_{z,\mathrm{d}}z}\right)\mathrm{e}^{\mathrm{i}(qx-\omega t)}\,\hat{\mathbf{y}}, \tag{6a}$$

$$\mathbf{E}_\mathrm{d} = \left[E_{x,\mathrm{d}}(z)\,\hat{\mathbf{x}} + E_{z,\mathrm{d}}(z)\,\hat{\mathbf{z}}\right]\mathrm{e}^{\mathrm{i}(qx-\omega t)}, \tag{6b}$$

in the dielectric half-space ($z > 0$), and

$$\mathbf{H}_\mathrm{m} = t_p^{(H)}\mathrm{e}^{-\mathrm{i}k_{z,\mathrm{m}}z}\mathrm{e}^{\mathrm{i}(qx-\omega t)}\,\hat{\mathbf{y}}, \tag{7a}$$

$$\mathbf{E}_\mathrm{m} = \left[E_{x,\mathrm{m}}(z)\,\hat{\mathbf{x}} + E_{z,\mathrm{m}}(z)\,\hat{\mathbf{z}}\right]\mathrm{e}^{\mathrm{i}(qx-\omega t)}, \tag{7b}$$

in the metal half-space ($z < 0$). Here $k_{z,j} = \sqrt{\epsilon_j k_0^2 - q^2}$ for $j \in \{\mathrm{d}, \mathrm{m}\}$. The explicit form of the amplitudes $E_{x/z,j}(z)$ readily follows from Maxwell's equations. Making use of the modified boundary conditions in Eqs. (5), one obtains the following linear system:

$$\frac{k_{z,\mathrm{d}}}{\epsilon_\mathrm{d}}\left(r_p^{(H)} - 1\right) + \frac{k_{z,\mathrm{m}}}{\epsilon_\mathrm{m}}t_p^{(H)} = \mathrm{i}q^2 d_\perp\left[\frac{1}{\epsilon_\mathrm{d}}\left(1 + r_p^{(H)}\right) - \frac{1}{\epsilon_\mathrm{m}}t_p^{(H)}\right], \tag{8a}$$

$$1 + r_p^{(H)} - t_p^{(H)} = -\mathrm{i}d_\parallel\left[k_{z,\mathrm{d}}(r_p^{(H)} - 1) + k_{z,\mathrm{m}}t_p^{(H)}\right]. \tag{8b}$$

After some algebraic manipulations, one finally arrives at the nonclassical Feibelman reflection and transmission coefficients for p-polarization (or TM polarization), which after changing to the Fresnel-like coefficients (i.e., defined in terms of the electric field), that is, $r^\mathrm{TM} \equiv r_p^{(H)}$ and $t^\mathrm{TM} \equiv \frac{\epsilon_\mathrm{d}k_{z,\mathrm{m}}}{\epsilon_\mathrm{m}k_{z,\mathrm{d}}}t_p^{(H)}$, read [9; 13]:

$$r^\mathrm{TM} = \frac{\epsilon_\mathrm{m}k_{z,\mathrm{d}} - \epsilon_\mathrm{d}k_{z,\mathrm{m}} + (\epsilon_\mathrm{m} - \epsilon_\mathrm{d})\left[\mathrm{i}q^2 d_\perp - \mathrm{i}k_{z,\mathrm{d}}k_{z,\mathrm{m}}d_\parallel\right]}{\epsilon_\mathrm{m}k_{z,\mathrm{d}} + \epsilon_\mathrm{d}k_{z,\mathrm{m}} - (\epsilon_\mathrm{m} - \epsilon_\mathrm{d})\left[\mathrm{i}q^2 d_\perp + \mathrm{i}k_{z,\mathrm{d}}k_{z,\mathrm{m}}d_\parallel\right]}, \tag{9a}$$

$$t^\mathrm{TM} = \frac{2\epsilon_\mathrm{d}k_{z,\mathrm{m}}}{\epsilon_\mathrm{m}k_{z,\mathrm{d}} + \epsilon_\mathrm{d}k_{z,\mathrm{m}} - (\epsilon_\mathrm{m} - \epsilon_\mathrm{d})\left[\mathrm{i}q^2 d_\perp + \mathrm{i}k_{z,\mathrm{d}}k_{z,\mathrm{m}}d_\parallel\right]}, \tag{9b}$$

where only terms up to linear order in $qd_{\perp,\parallel}$ have been included (for consistency with the assumptions made when computing the d-parameters [3]), and where $k_{z,j} = (\epsilon_j k_0^2 - q^2)^{1/2} = \mathrm{i}\kappa_j$ with $\kappa_j = (q^2 - \epsilon_j k_0^2)^{1/2}$ for $j \in \{\mathrm{d}, \mathrm{m}\}$.

Scattering coefficients for TM polarization in the nonretarded limit. In the nonretarded limit ($c \to \infty$) one has $k_{z,j} \to \mathrm{i}q$ and the reflection and transmission amplitudes reduce to their nonretarded forms [3]

$$r^\mathrm{TM,nr} = \frac{\epsilon_\mathrm{m} - \epsilon_\mathrm{d} + (\epsilon_\mathrm{m} - \epsilon_\mathrm{d})q\left[d_\perp + d_\parallel\right]}{\epsilon_\mathrm{m} + \epsilon_\mathrm{d} - (\epsilon_\mathrm{m} - \epsilon_\mathrm{d})q\left[d_\perp - d_\parallel\right]}, \tag{10a}$$

$$t^\mathrm{TM,nr} = \frac{2\epsilon_\mathrm{d}}{\epsilon_\mathrm{m} + \epsilon_\mathrm{d} - (\epsilon_\mathrm{m} - \epsilon_\mathrm{d})q\left[d_\perp - d_\parallel\right]}. \tag{10b}$$

Nonclassical reflection and transmission coefficients for TE waves (*s*-polarization)

The derivation of the nonclassical reflection and transmission coefficient for TE solutions (s-polarization) follows the same lines as the one detailed above for the TM polarization. In particular, the former are given by

$$r^{\mathrm{TE}} = \frac{k_{z,\mathrm{d}} - k_{z,\mathrm{m}} + (\epsilon_\mathrm{m} - \epsilon_\mathrm{d})\mathrm{i}k_0^2 d_\parallel}{k_{z,\mathrm{d}} + k_{z,\mathrm{m}} - (\epsilon_\mathrm{m} - \epsilon_\mathrm{d})\mathrm{i}k_0^2 d_\parallel}, \tag{11a}$$

$$t^{\mathrm{TE}} = \frac{2k_{z,\mathrm{d}}}{k_{z,\mathrm{d}} + k_{z,\mathrm{m}} - (\epsilon_\mathrm{m} - \epsilon_\mathrm{d})\mathrm{i}k_0^2 d_\parallel}. \tag{11b}$$

SUPPLEMENTARY NOTE 4. NONCLASSICAL SURFACE PLASMON POLARITONS: PLANAR INTERFACE

Equipped with the reflection coefficient for TM-polarization in Eq. (9a), the resulting surface plasmon-polariton (SPP) dispersion can be readily obtained from its poles. Hence, the implicit condition for the SPP dispersion relation including quantum surface corrections via Feibelman d-parameters is given by

$$\frac{\epsilon_\mathrm{d}}{\kappa_\mathrm{d}} + \frac{\epsilon_\mathrm{m}}{\kappa_\mathrm{m}} - (\epsilon_\mathrm{m} - \epsilon_\mathrm{d})\left[\frac{q^2}{\kappa_\mathrm{m}\kappa_\mathrm{d}} d_\perp - d_\parallel\right] = 0. \tag{12}$$

We stress that here $\epsilon_\mathrm{m} \equiv \epsilon_\mathrm{m}(\omega)$ is still the local bulk dielectric function of the metal, and therefore the nonclassical corrections (e.g., spill-out, nonlocality, electron-hole pair generation) are encoded and enter via the d-parameters alone. Naturally, it is apparent from Eq. (12) that the well-know classical result, $\epsilon_\mathrm{d}/\kappa_\mathrm{d} + \epsilon_\mathrm{m}/\kappa_\mathrm{m} = 0$, is reinstated upon letting $d_{\perp,\parallel} \to 0$.

Nonretarded surface plasmon dispersion for a planar interface. Taking the nonretarded limit of Eq. (12) leads to the following condition for the nonretarded surface plasmon dispersion:

$$\epsilon_\mathrm{m} + \epsilon_\mathrm{d} - (\epsilon_\mathrm{m} - \epsilon_\mathrm{d})q\,[d_\perp - d_\parallel] = 0. \tag{13}$$

Clearly, contrary to the corresponding classical case, i.e., $\epsilon_\mathrm{m} = -\epsilon_\mathrm{d}$, the surface plasmon spectrum is not dispersionless anymore, even in the nonretarded regime. Indeed, one now may also write instead:

$$q_\mathrm{sp} = \frac{\epsilon_\mathrm{m} + \epsilon_\mathrm{d}}{\epsilon_\mathrm{m} - \epsilon_\mathrm{d}} \frac{1}{d_\perp - d_\parallel}. \tag{14}$$

Yet, writing an explicit solution in closed-form for the surface plasmon frequency as a function of wavevector, i.e., ω *vs.* q, is not possible due to the implicit frequency dependence of the d-parameters. Nevertheless, starting from Eq. (13), one may work towards a perturbative solution for the SPP (complex) eigenfrequencies.

In particular, within the nonretarded limit, and for a Drude-like dielectric function $\epsilon_\mathrm{m}(\omega) = \epsilon_\infty - \omega_\mathrm{p}^2/(\omega^2 + \mathrm{i}\omega\gamma)$, approximate expressions for the real and imaginary parts of the first-order spectral correction, $\omega = \omega^{\mathrm{cl}} + \omega^{(1)} + O([\omega - \omega^{\mathrm{cl}}]^2)$, can be obtained. Then, assuming that $\mathrm{Re}\,\omega \gg \gamma$ and $qd_{\perp,\parallel} \ll 1$, we have

$$\mathrm{Re}\,\omega \simeq \omega_\mathrm{sp}^{(0)} - \omega_\mathrm{sp}^{(0)} \frac{\epsilon_\mathrm{d}}{\epsilon_\mathrm{d} + \epsilon_\infty} \underbrace{q\,\mathrm{Re}\left(d_\perp^{(0)} - d_\parallel^{(0)}\right)}_{\substack{\text{electronic}\\ \text{spill-out/spill-in}}}, \tag{15a}$$

$$-\mathrm{Im}\,\omega \simeq \underbrace{\frac{\gamma}{2}}_{\substack{\text{classical}\\ \text{bulk}\\ \text{damping}}} + \omega_\mathrm{sp}^{(0)} \frac{\epsilon_\mathrm{d}}{\epsilon_\mathrm{d} + \epsilon_\infty} \underbrace{q\,\mathrm{Im}\left(d_\perp^{(0)} - d_\parallel^{(0)}\right)}_{\substack{\text{surface-enabled}\\ \text{Landau damping}}}, \tag{15b}$$

where $\omega_\mathrm{sp}^{(0)} \equiv \mathrm{Re}\,\omega^{\mathrm{cl}} = \omega_\mathrm{p}/\sqrt{\epsilon_\infty + \epsilon_\mathrm{d}}$ is the classical nonretarded surface plasmon frequency. Also, $d_{\perp,\parallel}^{(0)} \equiv d_{\perp,\parallel}(\omega_\mathrm{sp}^{(0)})$ is a pole-like approximation (necessary here owing to the explicit frequency dependence of

the d-parameters). Equations (15b) underscore that the direction (magnitude) of the nonclassical shift of the SPP resonance depends on the sign (magnitude) of the real part of $d_{\text{eff}} \equiv d_\perp - d_\parallel$; and, that the nonclassical SPP resonance broadening is proportional to the imaginary part of d_{eff}.

Notably, for the instructive case of a homogeneous three-dimensional electron gas (3DEG) (i.e., in which $\epsilon_\infty = 1$ and $d_\parallel = 0$) with $\epsilon_{\text{d}} = 1$, Eqs. (15b) take a particularly compact form, reading

$$\operatorname{Re}\omega \simeq \omega_{\text{sp}}^{(0)}\left[1 - \frac{q}{2}\operatorname{Re}\left(d_\perp^{(0)}\right)\right] \quad \text{and} \quad \operatorname{Im}\omega \simeq -\frac{\gamma}{2}\left[1 + \frac{\omega_{\text{sp}}^{(0)}}{\gamma} q \operatorname{Im}\left(d_\perp^{(0)}\right)\right], \tag{16}$$

which reproduces the result obtained by Feibelman [9], and, more recently, by Christensen *et al.* [3]. At this point, the reader may appreciate that, despite being only approximate, Eqs. (15b) deliver a clear message: to lowest order, the direction of the frequency shift due to nonlocal quantum surface phenomena ultimately depends on the sign of $\operatorname{Re}(d_\perp - d_\parallel)$. Specifically, for a fixed frequency, these nonclassical corrections lead to redshift (blueshift) of the surface plasmon resonance—relative to the classical case—if $\operatorname{Re}(d_\perp - d_\parallel) > 0$ (< 0). Furthermore, for charge-neutral surfaces so that $d_\parallel = 0$, and recalling the interpretation of $d_\perp$ as the centroid of the induced charge density, then one may associate the positiveness or negativeness of $d_\perp$, respectively, to a "spill-out" or a "spill-in" of the induced electron density with respect to the fixed ionic background of the metal.

SPP dispersion for $r_s = 2$ jellium and Ag. Figure 3 depicts the quantum mechanical SPP dispersion diagram for a planar interface between air and a jellium metal with $r_s = 2$ (representative of Al), together with the SPP dispersion for a flat air–silver interface. The corresponding classical spectral properties are also

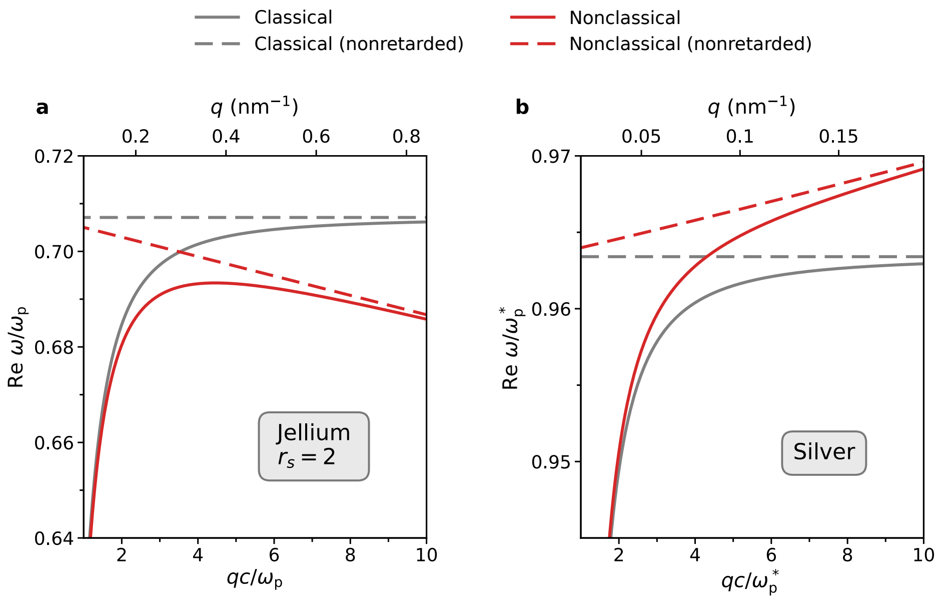

Supplementary Figure 3. **a** Quantum corrections via Feibelman d-parameters to the SPP's dispersion for a flat air–jellium interface ($r_s = 2$ jellium with Drude-type damping $\hbar\gamma = 0.283$ eV; so that the ratio γ/ω_{p} is the same as in the $r_s = 4$ case presented in the main text). **b** Classical and quantum mechanical dispersion relations for SPPs in a air–Ag interface. We model silver with a bulk dielectric function $\epsilon_{\text{m}}(\omega) = \epsilon_\infty(\omega) - \omega_{\text{p}}^2/(\omega^2 + i\omega\gamma)$ and use the d-parameters data outlined in Supplementary Figure 2 to account for the quantum mechanical effects discussed in the text. The background permittivity of silver is obtained from Johnson and Christy's experimental data [16] via $\epsilon_\infty(\omega) = \epsilon_{\text{exp}}(\omega) + \omega_{\text{p}}^2/(\omega^2 + i\omega\gamma)$. We use $\hbar\omega_{\text{p}} = 9.02$ eV and $\hbar\gamma = 22$ meV (which fits well the experimental data). The screened plasma frequency is $\hbar\omega_{\text{p}}^* = 3.81$ eV.

shown to facilitate the comparison and interpretation of the nonclassical results. As the figure indicates, we predict a nonclassical redshift (blueshift) of the SPP resonance, consistent with the fact that $\operatorname{Re} d_{\text{eff}} > 0$ ($\operatorname{Re} d_{\text{eff}} < 0$) in the relevant frequency ranges. Thus, the observed redshift of the SPP frequency for jellium metals is a consistent with the of the quantum mechanical spill-out of the induced electron density beyond the classically-forbidden jellium edge [4; 17–20]. Conversely, for noble metals like silver, $\operatorname{Re} d_{\text{eff}} < 0$

around $\omega^{\rm cl}$ due to d-band screening [3; 4]. Therefore, and contrary to the jellium case, here we predict a blueshift of the SPP resonances, in line with experimental observations [20–24]. This can be readily seen upon inspection of Supplementary Figure 3b which shows a blueshift of the SPP eigenfrequencies for large wavevectors.

SUPPLEMENTARY NOTE 5. GENERALIZED MIE THEORY WITH FEIBELMAN *d*-PARAMETERS: SPHERICAL METAL PARTICLES

In what follows we develop a generalized Mie theory that includes nonlocal and quantum mechanical effects incorporated via Feibelman d-parameters. As in the planar case, the possession of such quantum Mie coefficients is all that is required in order to readily determine the sphere's localized plasmon resonances, scattering cross-sections, Purcell enhancements, etc.

Theoretical framework

Wave equation in spherically symmetric systems. In a linear, isotropic and homogeneous medium the electric and magnetic fields must satisfy their respective vector wave equations,

$$\nabla^2 \mathbf{E}(\mathbf{r},\omega) + k_j^2 \mathbf{E}(\mathbf{r},\omega) = 0 \quad \text{and} \quad \nabla^2 \mathbf{H}(\mathbf{r},\omega) + k_j^2 \mathbf{H}(\mathbf{r},\omega) = 0, \tag{17}$$

where $k_j \equiv \sqrt{\epsilon_j} k_0$. The electric and magnetic fields in spherical coordinates can be constructed in terms of vector harmonics, $\mathbf{M}_\nu(\mathbf{r})$ and $\mathbf{N}_\nu(\mathbf{r})$, which in turn are defined in terms of a pilot wave $\mathbf{c}$ and a generating scalar function $\psi_\nu(\mathbf{r})$ that obeys the (scalar) Helmholtz equation $\nabla^2 \psi_\nu(\mathbf{r}) + k_j^2 \psi_\nu(\mathbf{r}) = 0$ [25]. Strictly speaking, the pilot vector $\mathbf{c}$ should be a constant vector. However, and rather fortunately, in spherical coordinates and in this case alone, it turns out that we can take $\mathbf{c} = r\hat{\mathbf{r}}$; this enables us to construct a tangential solution $\mathbf{M}_\nu(\mathbf{r})$ (though $\mathbf{N}_\nu(\mathbf{r})$ is not purely normal) [26]. This is desirable because it allows us to associate each of the vector harmonics $\mathbf{M}_\nu(\mathbf{r})$ and $\mathbf{N}_\nu(\mathbf{r})$ with TE and TM waves, respectively.

Therefore, the vector harmonics $\mathbf{M}_\nu(\mathbf{r})$ and $\mathbf{N}_\nu(\mathbf{r})$—which are two independent solutions of the vector wave equation in spherical coordinates—may be written as [25; 26]

$$\mathbf{M}_{lm}^{e/o}(\mathbf{r}) \equiv \nabla \times \hat{\mathbf{r}}\, r \psi_{lm}^{e/o}(\mathbf{r}), \tag{18a}$$

$$\mathbf{N}_{lm}^{e/o}(\mathbf{r}) \equiv k_j^{-1} \nabla \times \nabla \times \hat{\mathbf{r}}\, r \psi_{lm}^{e/o}(\mathbf{r}), \tag{18b}$$

where the generation function is

$$\psi_{lm}^{e/o}(r,\theta,\phi) \equiv z_l(k_j r) P_l^m(\cos\theta) \left\{ \begin{array}{c} \cos(m\phi) \\ \sin(m\phi) \end{array} \right\}, \tag{18c}$$

and where the radial part $z_l(k_j r)$ is either a spherical Bessel or Hankel function of the first kind, $j_l(k_j r)$ and $h_l^{(1)}(k_j r)$, respectively representing incoming and outgoing waves. Furthermore, the "quantum numbers" l and m are integers with values in the range $l \in [1, \infty[$ and $m \in [0, l]$, and P_l^m denote the associated Legendre polynomials. The explicit form of the solenoidal (i.e., divergence-free) vector waves $\mathbf{M}_{lm}^{e/o}(\mathbf{r})$

and $\mathbf{N}_{lm}^{e/o}(\mathbf{r})$, obtained via Eqs. (18), reads[3]

$$\mathbf{M}_{lm}^{e/o}(\mathbf{r}) = \begin{bmatrix} 0 \\ m z_l(\rho_j)\dfrac{P_l^m(\mu)}{\sin\theta}\left\{\begin{array}{c} -\sin(m\phi) \\ \cos(m\phi)\end{array}\right\} \\ -z_l(\rho)\dfrac{\partial P_l^m(\mu)}{\partial\theta}\left\{\begin{array}{c}\cos(m\phi)\\ \sin(m\phi)\end{array}\right\}\end{bmatrix} \quad \text{and} \quad \mathbf{N}_{lm}^{e/o}(\mathbf{r}) = \begin{bmatrix} \dfrac{l(l+1)}{\rho_j} z_l(\rho_j) P_l^m(\mu)\left\{\begin{array}{c}\cos(m\phi)\\ \sin(m\phi)\end{array}\right\} \\ \dfrac{1}{\rho_j}\left[\rho_j z_l(\rho_j)\right]' \dfrac{\partial P_l^m(\mu)}{\partial\theta}\left\{\begin{array}{c}\cos(m\phi)\\ \sin(m\phi)\end{array}\right\} \\ \dfrac{m}{\rho_j}\left[\rho_j z_l(\rho_j)\right]' \dfrac{P_l^m(\mu)}{\sin\theta}\left\{\begin{array}{c}-\sin(m\phi)\\ \cos(m\phi)\end{array}\right\}\end{bmatrix}, \quad (19)$$

where $\rho_j \equiv k_j r$ and $\mu \equiv \cos\theta$ (for the sake of brevity of notation), and the prime denotes differentiation with respect to ρ_j.

The spherical vector harmonics $\mathbf{M}_{lm}^{e/o}(\mathbf{r})$ and $\mathbf{N}_{lm}^{e/o}(\mathbf{r})$ may be regarded as fundamental solutions of the vector wave equation in spherical coordinates. Hence, the electric and magnetic fields can be constructed from an expansion in terms of such vector waves.

Incident plane-wave in terms of vector spherical harmonics. Let us assume an incident plane-wave traveling along the positive z-direction and polarized along $\hat{\mathbf{x}}$, namely[4]

$$\mathbf{E}^{\text{inc}} = E_0 e^{ik_d z}\hat{\mathbf{x}} = E_0 e^{ik_d r\cos\theta}\begin{bmatrix}\sin\theta\cos\phi \\ \cos\theta\cos\phi \\ -\sin\theta\end{bmatrix}. \quad (20)$$

We now want to cast the incident field, not as in Eq. (20), but rather in terms of the vector spherical waves introduced earlier in the text, that is

$$\mathbf{E}^{\text{inc}} = \sum_{l=1}^{\infty}\sum_{m=0}^{l}\sum_{\sigma=\{e,o\}}\left(A_{lm}^{\text{TM},\sigma}\mathbf{N}_{lm}^{\sigma} + A_{lm}^{\text{TE},\sigma}\mathbf{M}_{lm}^{\sigma}\right), \quad (21)$$

where the expansion coefficients $A_{lm}^{\text{TM},\sigma}$ and $A_{lm}^{\text{TE},\sigma}$ follow from [25]

$$A_{lm}^{\text{TM},\sigma} = \frac{\int_0^{2\pi} d\phi \int_0^{\pi} d\theta \sin\theta\, \mathbf{E}^{\text{inc}}\cdot\mathbf{N}_{lm}^{\sigma}}{\int_0^{2\pi} d\phi \int_0^{\pi} d\theta \sin\theta \left|\mathbf{N}_{lm}^{\sigma}\right|^2} \quad \text{and} \quad A_{lm}^{\text{TE},\sigma} = \frac{\int_0^{2\pi} d\phi \int_0^{\pi} d\theta \sin\theta\, \mathbf{E}^{\text{inc}}\cdot\mathbf{M}_{lm}^{\sigma}}{\int_0^{2\pi} d\phi \int_0^{\pi} d\theta \sin\theta \left|\mathbf{M}_{lm}^{\sigma}\right|^2}. \quad (22)$$

Carrying out the explicit calculations and exploiting the orthogonality relations of the sine and cosines functions, one finds that $A_{lm}^{\text{TM},o} = 0$ and $A_{lm}^{\text{TM},e} \propto \delta_{m1}$, and, similarly, that $A_{lm}^{\text{TE},e} = 0$ and $A_{lm}^{\text{TE},o} \propto \delta_{m1}$. The calculation of these coefficients then leads to

$$\mathbf{E}^{\text{inc}} = \sum_{l=1}^{\infty} E_0^l\left(-i\mathbf{N}_{l1}^{e\,[d]} + \mathbf{M}_{l1}^{o\,[d]}\right), \quad (23)$$

where the extra superscript "[d]" indicates that the radial function is $z_l(\rho_d) \equiv j_l(\rho_d)$ because the impinging field has to remain finite at the origin, and we have also defined $E_0^l = i^l \frac{2l+1}{l(l+1)} E_0$. From here, the corresponding incident magnetic field follows from Faraday's law, yielding

$$\mathbf{H}^{\text{inc}} = -\frac{k_d}{\omega\mu_0}\sum_{l=1}^{\infty} E_0^l\left(i\mathbf{N}_{l1}^{o\,[d]} + \mathbf{M}_{l1}^{e\,[d]}\right), \quad (24)$$

where the properties of the vector spherical harmonics have been used [25; 26].

Equations (23) and (24) thereby reflect the expansion of the incident plane-wave in terms of vector spherical waves.

---

[3] For the radial component of $\mathbf{N}_{lm}^{e/o}(\mathbf{r})$ we have made use of the differential Legendre equation whose solutions are the associated Legendre polynomials.
[4] Note that $\hat{\mathbf{x}} = \sin\theta\cos\phi\,\hat{\mathbf{r}} + \cos\theta\cos\phi\,\hat{\boldsymbol{\theta}} - \sin\theta\,\hat{\boldsymbol{\phi}}$.

Internal and scattered fields. In possession of the incident field expansion, Eqs. (23) and (24), we now express in a similar fashion the internal and scattered fields using vector spherical waves. In this spirit, we write the fields inside the sphere as

$$\mathbf{E}^{\mathrm{m}} = \sum_{l=1}^{\infty} E_0^l \left(-\mathrm{i} c_l^{\mathrm{TM}} \mathbf{N}_{l1}^{e\,[\mathrm{m}]} + c_l^{\mathrm{TE}} \mathbf{M}_{l1}^{o\,[\mathrm{m}]} \right), \tag{25a}$$

$$\mathbf{H}^{\mathrm{m}} = -\frac{k_{\mathrm{m}}}{\omega \mu_0} \sum_{l=1}^{\infty} E_0^l \left(\mathrm{i} c_l^{\mathrm{TE}} \mathbf{N}_{l1}^{o\,[\mathrm{m}]} + c_l^{\mathrm{TM}} \mathbf{M}_{l1}^{e\,[\mathrm{m}]} \right). \tag{25b}$$

where now the superscript "[m]" highlights that the radial function is $z_l(\rho_{\mathrm{m}}) \equiv j_l(\rho_{\mathrm{m}})$ because it needs to remain finite at the origin.

Outside the sphere, both spherical Bessel functions j_l and y_l are permitted and therefore we shall use the spherical Hankel function of the first kind for the radial part of the generating function. Specifically, we choose $z_l(\rho_{\mathrm{d}}) \equiv h_l^{(1)}(\rho_{\mathrm{d}})$ owing to its appropriate asymptotic behavior at large ρ_{d} [25], i.e., corresponding to an outgoing spherical wave (which is what we expect for the asymptotic scattered field). Within this reasoning, we therefore express the scattered fields as

$$\mathbf{E}^{\mathrm{scat}} = \sum_{l=1}^{\infty} E_0^l \left(\mathrm{i} a_l^{\mathrm{TM}} \mathbf{N}_{l1}^{e\,[\mathrm{s}]} - a_l^{\mathrm{TE}} \mathbf{M}_{l1}^{o\,[\mathrm{s}]} \right), \tag{26a}$$

$$\mathbf{H}^{\mathrm{scat}} = \frac{k_{\mathrm{d}}}{\omega \mu_0} \sum_{l=1}^{\infty} E_0^l \left(\mathrm{i} a_l^{\mathrm{TE}} \mathbf{N}_{l1}^{o\,[\mathrm{s}]} + a_l^{\mathrm{TM}} \mathbf{M}_{l1}^{e\,[\mathrm{s}]} \right), \tag{26b}$$

where this time the superscript "[s]" indicates that the radial function is $h_l^{(1)}(\rho_{\mathrm{d}})$ as we have just mentioned.

The amplitudes a_l^{TM}, a_l^{TE}, c_l^{TM}, and c_l^{TE}, are the so-called Mie coefficients, of the scattered and internal (i.e. transmitted) kind, in TM and TE flavors, respectively. These can be determined once the adequate boundary conditions are imposed (at the sphere's surface).

Nonclassical Mie coefficients

The Mie coefficients essentially entail all the relevant physics describing the electromagnetic response of a sphere of arbitrary size and material constitution. In the following, we derive closed-form expressions for the generalized (i.e., nonclassical) Mie coefficients within the formalism of Feibelman d-parameters. In order to proceed, we need to invoke the appropriate boundary conditions, reading (cf. SUPPLEMENTARY NOTE 2), in spherical coordinates,

$$\left(\mathbf{E}_{\Omega}^{\mathrm{inc}} + \mathbf{E}_{\Omega}^{\mathrm{scat}} - \mathbf{E}_{\Omega}^{\mathrm{m}} \right)\Big|_{r=R} = -d_{\perp} \nabla_{\Omega} \left(E_r^{\mathrm{inc}} + E_r^{\mathrm{scat}} - E_r^{\mathrm{m}} \right)\Big|_{r=R}, \tag{27a}$$

$$\left(\mathbf{H}_{\Omega}^{\mathrm{inc}} + \mathbf{H}_{\Omega}^{\mathrm{scat}} - \mathbf{H}_{\Omega}^{\mathrm{m}} \right)\Big|_{r=R} = \mathrm{i}\omega d_{\parallel} \left(\mathbf{D}_{\Omega}^{\mathrm{inc}} + \mathbf{D}_{\Omega}^{\mathrm{scat}} - \mathbf{D}_{\Omega}^{\mathrm{m}} \right) \times \hat{\mathbf{r}}\Big|_{r=R}, \tag{27b}$$

with $\Omega = \{\theta, \phi\}$ denoting the angular components (i.e., the components that are tangential to the sphere's surface). Equations (27), after some algebra, produce the following set of equations:

$$h_l^{(1)}(x_{\mathrm{d}}) a_l^{\mathrm{TE}} + j_l(x_{\mathrm{m}}) c_l^{\mathrm{TE}} = j_l(x_{\mathrm{d}}), \tag{28a}$$

$$\left[\xi_l'(x_{\mathrm{d}}) + \overline{d}_{\perp} h_l^{(1)}(x_{\mathrm{d}}) \right] a_l^{\mathrm{TM}} + \left[\Psi_l'(x_{\mathrm{m}}) + \overline{d}_{\perp} j_l(x_{\mathrm{m}}) \right] \frac{k_{\mathrm{d}}}{k_{\mathrm{m}}} c_l^{\mathrm{TM}} = \Psi_l'(x_{\mathrm{d}}) + \overline{d}_{\perp} j_l(x_{\mathrm{d}}), \tag{28b}$$

$$\left[h_l^{(1)}(x_{\mathrm{d}}) + \overline{d}_{\parallel} \xi_l'(x_{\mathrm{d}}) \right] a_l^{\mathrm{TM}} + \left[j_l(x_{\mathrm{m}}) + \overline{d}_{\parallel} \Psi_l'(x_{\mathrm{m}}) \right] \frac{k_{\mathrm{m}}}{k_{\mathrm{d}}} c_l^{\mathrm{TM}} = j_l(x_{\mathrm{d}}) + \overline{d}_{\parallel} \Psi_l'(x_{\mathrm{d}}), \tag{28c}$$

$$\left[\xi_l'(x_{\mathrm{d}}) - x_{\mathrm{d}}^2 \overline{d}_{\parallel} h_l^{(1)}(x_{\mathrm{d}}) \right] a_l^{\mathrm{TE}} + \left[\Psi_l'(x_{\mathrm{m}}) - x_{\mathrm{m}}^2 \overline{d}_{\parallel} j_l(x_{\mathrm{m}}) \right] c_l^{\mathrm{TE}} = \Psi_l'(x_{\mathrm{d}}) - x_{\mathrm{d}}^2 \overline{d}_{\parallel} j_l(x_{\mathrm{d}}), \tag{28d}$$

with $x_j = k_j R$. Additionally, the Riccati-Bessel functions $\Psi_l(x) = x j_l(x)$ and $\xi_l(x) = x h_l^{(1)}(x)$ have been employed, and the prime denotes the functions' derivatives with respect to their argument. Lastly, for

shorthand notation, we have also introduced the dimensionless quantities

$$\overline{d}_\perp \equiv l(l+1)\frac{d_\perp}{R} \qquad \text{and} \qquad \overline{d}_\parallel \equiv \frac{d_\parallel}{R}. \tag{29a}$$

Solving the system of equations posed by Eqs. (28), we obtain the mesoscopic generalization of the Mie coefficients incorporating the Feibelman d-parameters:

$$a_l^{\text{TM}} = \frac{\epsilon_{\text{m}} j_l(x_{\text{m}})\Psi_l'(x_{\text{d}}) - \epsilon_{\text{d}} j_l(x_{\text{d}})\Psi_l'(x_{\text{m}}) + (\epsilon_{\text{m}} - \epsilon_{\text{d}})\left[j_l(x_{\text{d}})j_l(x_{\text{m}})\overline{d}_\perp + \Psi_l'(x_{\text{d}})\Psi_l'(x_{\text{m}})\overline{d}_\parallel\right]}{\epsilon_{\text{m}} j_l(x_{\text{m}})\xi_l'(x_{\text{d}}) - \epsilon_{\text{d}} h_l^{(1)}(x_{\text{d}})\Psi_l'(x_{\text{m}}) + (\epsilon_{\text{m}} - \epsilon_{\text{d}})\left[h_l^{(1)}(x_{\text{d}})j_l(x_{\text{m}})\overline{d}_\perp + \xi_l'(x_{\text{d}})\Psi_l'(x_{\text{m}})\overline{d}_\parallel\right]}, \tag{30a}$$

$$a_l^{\text{TE}} = \frac{j_l(x_{\text{m}})\Psi_l'(x_{\text{d}}) - j_l(x_{\text{d}})\Psi_l'(x_{\text{m}}) + (x_{\text{m}}^2 - x_{\text{d}}^2)j_l(x_{\text{d}})j_l(x_{\text{m}})\overline{d}_\parallel}{j_l(x_{\text{m}})\xi_l'(x_{\text{d}}) - h_l^{(1)}(x_{\text{d}})\Psi_l'(x_{\text{m}}) + (x_{\text{m}}^2 - x_{\text{d}}^2)h_l^{(1)}(x_{\text{d}})j_l(x_{\text{m}})\overline{d}_\parallel}, \tag{30b}$$

for the coefficients associated with the scattered outgoing wave, and

$$c_l^{\text{TE}} = \frac{j_l(x_{\text{d}})\xi_l'(x_{\text{d}}) - h_l^{(1)}(x_{\text{d}})\Psi_l'(x_{\text{d}})}{j_l(x_{\text{m}})\xi_l'(x_{\text{d}}) - h_l^{(1)}(x_{\text{d}})\Psi_l'(x_{\text{m}}) + (x_{\text{m}}^2 - x_{\text{d}}^2)h_l^{(1)}(x_{\text{d}})j_l(x_{\text{m}})\overline{d}_\parallel}, \tag{31a}$$

$$c_l^{\text{TM}} = \sqrt{\epsilon_{\text{m}}\epsilon_{\text{d}}}\,\frac{j_l(x_{\text{d}})\xi_l'(x_{\text{d}}) - h_l^{(1)}(x_{\text{d}})\Psi_l'(x_{\text{d}})}{\epsilon_{\text{m}} j_l(x_{\text{m}})\xi_l'(x_{\text{d}}) - \epsilon_{\text{d}} h_l^{(1)}(x_{\text{d}})\Psi_l'(x_{\text{m}}) + (\epsilon_{\text{m}} - \epsilon_{\text{d}})\left[h_l^{(1)}(x_{\text{d}})j_l(x_{\text{m}})\overline{d}_\perp + \xi_l'(x_{\text{d}})\Psi_l'(x_{\text{m}})\overline{d}_\parallel\right]}, \tag{31b}$$

for the coefficients associated with the internal fields. As in the planar case, we have only kept terms up to linear order in the d-parameters. We have also added TM and TE as superscripts in the Mie coefficients to highlight the character of the associated waves.

Notice that the quantum mechanical Mie coefficients a_l^{TE} and c_l^{TE}, which describe TE waves, are independent of the perpendicular Feibelman d-parameter, $d_\perp$. This should not really constitute a surprise, since for a TE wave the electric field is purely tangential to the particle's surface and therefore has no perpendicular component capable of inducing a displacement of the charge density along the perpendicular to the sphere's surface. On the other hand, the Mie coefficients a_l^{TM} and c_l^{TM}, associated to TM waves, and thus the electric field in general possesses both radial and tangential components, and consequently both $d_\perp$ and $d_\parallel$ emerge in these coefficients.

To the best of our knowledge, such quantum mechanical Mie coefficients with the incorporation of nonlocal and quantum surface corrections via Feibelman d-parameters, given above by Eqs. (30) and (31), have not been derived before. Thus, these results could open new perspectives for the investigation of nonlocal and quantum effects in the context of light–matter interactions in nanophotonics with metal spheres. With this in mind, they constitute a springboard to study phenomena beyond the classical regime, namely the influence of quantum nonlocal effects on the cross-sections, localized surface plasmon (LSP) resonances, electromagnetic LDOS, etc, for spherical metal particles of arbitrary size.

Nonretarded multipolar polarizability and localized surface plasmons. In the nonretarded limit, the outgoing Mie coefficients reduce to a single quantity: the nonretarded multipolar polarizability, α_l. Since in the nonretarded regime we have $\omega R/c \to 0$, then one may perform small-argument expansions of $j_l(z)$, $h_l^{(1)}(z)$, and of the derivatives of the Riccati-Bessel functions, thereby yielding the following relation between the Mie coefficient a_l^{TM} and the multipolar polarizability:

$$\lim_{\omega R/c \to 0} a_l^{\text{TM}} = -\text{i}x_{\text{d}}^{2l+1}\frac{(l+1)(2l+1)}{l\,[(2l+1)!!]^2}\frac{\alpha_l}{4\pi R^{2l+1}}, \tag{32}$$

where

$$\alpha_l = 4\pi R^{2l+1}\frac{(\epsilon_{\text{m}} - \epsilon_{\text{d}})\left[1 + \frac{l}{R}\left(d_\perp + \frac{l+1}{l}d_\parallel\right)\right]}{\epsilon_{\text{m}} + \frac{l+1}{l}\epsilon_{\text{d}} - (\epsilon_{\text{m}} - \epsilon_{\text{d}})\frac{l+1}{R}(d_\perp - d_\parallel)}. \tag{33}$$

Then, the nonretarded localized surface plasmon resonances immediately stem from the poles of α_l, reading

$$\epsilon_{\text{m}} + \frac{l+1}{l}\epsilon_{\text{d}} - (\epsilon_{\text{m}} - \epsilon_{\text{d}})\frac{l+1}{R}(d_\perp - d_\parallel) = 0. \tag{34}$$

In the limit of vanishing d-parameters, Eqs. (33) and (34) appropriately reduce to their well-known classical equivalents, that is, $\alpha_l^{\text{cl}} = 4\pi R^{2l+1} \dfrac{\epsilon_{\text{m}} - \epsilon_{\text{d}}}{\epsilon_{\text{m}} + \epsilon_{\text{d}}(l+1)/l}$ and $\epsilon_{\text{m}} + \dfrac{l+1}{l}\epsilon_{\text{d}} = 0$, respectively.

Optical response of plasmonic spheres

A particularly alluring feature of the theoretical formalism set forth here, it that the mathematical structure of the usual expressions for the cross-sections, LDOS, etc, is unchanged; that is, the nonclassical optical response is obtained using the very same textbook expressions, simply by replacing the classical Mie coefficients by the quantum mechanical Mie coefficients presented in Eqs. (30). Hence, for instance, the extinction cross-section for a metal sphere illuminated by a monochromatic plane-wave is therefore given by

$$\sigma_{\text{ext}} = \frac{2\pi}{k_{\text{d}}^2} \sum_{l=1}^{\infty} (2l+1)\, \text{Re}\{a_l^{\text{TM}} + a_l^{\text{TE}}\}, \tag{35}$$

For relatively small metal spheres, the optical response is dominated by a series of peaks originating from the excitation of LSP resonances. The latter occur at frequencies for which a_l^{TM} has pole, as illustrated in Supplementary Figure 4. A LSP resonance is said to be of electric dipole character for $l = 1$, of electric quadrupole character for $l = 2$, and so on. It is also apparent from the figure that the dipole resonance ($l = 1$) contributes the most for the cross-section, with the successively higher-order resonances becoming increasingly negligible (notice the logarithmic scale). The TE Mie coefficients a_l^{TE} are essentially featureless and their contribution for the extinction cross-section is several orders of magnitude smaller than the one associated with TM Mie coefficients a_l^{TM} of the same order. The impact of quantum nonlocal effects, i.e., a redshift owing to spill-out ($\text{Re}\, d_\perp > 0$) and broadening due to Landau damping, can be clearly observed in Supplementary Figure 4 as well. It is also interesting to note that the TE Mie coefficients are unchanged by such effects, which can be understood by the fact that here $d_\parallel = 0$ [recall Eq. (30b)].

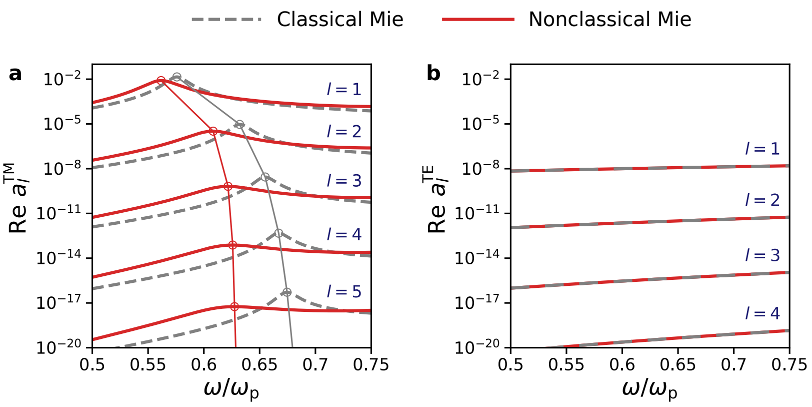

Supplementary Figure 4. Real parts of the Mie coefficients for a $R = 5$ nm jellium sphere with $r_s = 4$. The metal particle is assumed to be embedded in vacuum (ϵ_{d}=1), the d-parameters are shown in Figure 1 of the main paper, and a Drude-type broadening of $\hbar\gamma = 0.1$ eV is assumed. **a** Real part of the Mie coefficient associated with TM polarization, a_l^{TM}, for different angular momentum, l (the line-connected circles indicate their respective maxima). **b** Real part of the Mie coefficient associated with TM polarization, a_l^{TE}, for different angular momentum, l.

In the nonretarded regime, the TM Mie coefficient a_l^{TM} reduces to

$$\lim_{\omega R/c \to 0} a_l^{\text{TM}} = -i x_{\text{d}}^{2l+1} \frac{(l+1)(2l+1)}{l\,[(2l+1)!!]^2} \frac{\alpha_l}{4\pi R^{2l+1}}\,. \tag{36}$$

and σ_{ext} is completely characterized by the nonretarded multipolar polarizability, α_l.

In Supplementary Figure 5 we plot the normalized extinction cross-sections, $\sigma_{\text{ext}}/(\pi R^2)$, for $r_s = 4$ jellium spheres of different radii. As expected, although the predictions from classical electrodynamics suffice for

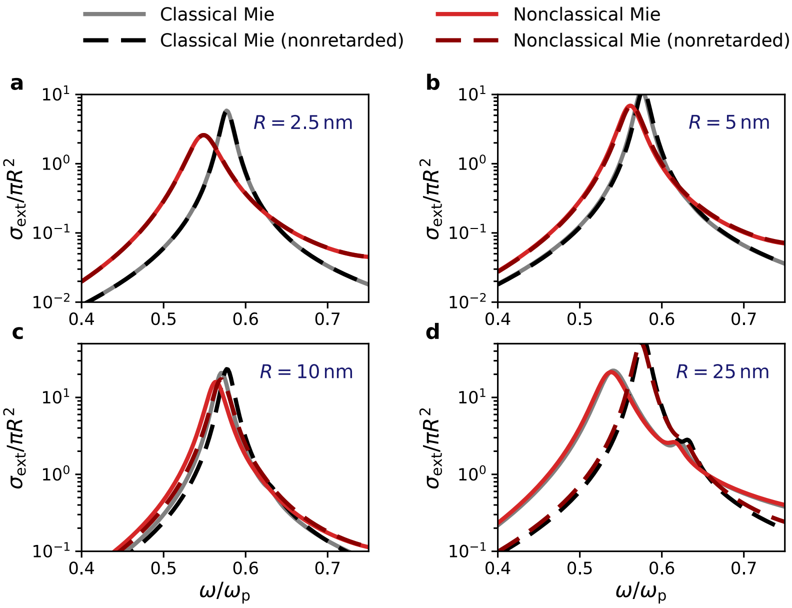

Supplementary Figure 5. Normalized extinction cross-sections, $\sigma_{\text{ext}}/(\pi R^2)$, for $r_s = 4$ jellium spheres of different radius. The parameters for the metal are the same as in Supplementary Figure 4. **a** $R = 2.5\,\text{nm}$; **b** $R = 5\,\text{nm}$; **c** $R = 10\,\text{nm}$; **d** $R = 25\,\text{nm}$.

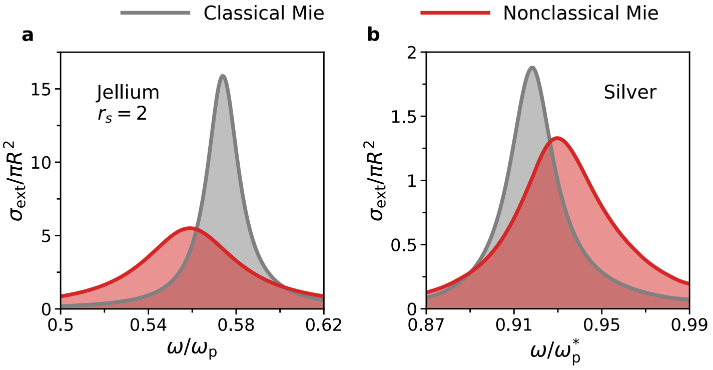

Supplementary Figure 6. Normalized extinction cross-section for $R = 2.5\,\text{nm}$ metal spheres made of **a** $r_s = 2$ jellium; and **b** silver. The parameters are the same as in Supplementary Figure 3.

large spheres, they rapidly decline for the spheres of smaller radii. Moreover, the figure also underscores the importance of retardation effects for metal spheres that are small, but not *too small*. We end the discussion of the far-field optical response of plasmonic spheres by comparing the extinction cross-section of a $r_s = 2$ jellium sphere with the one of a silver nanoparticle. The results are depicted in Supplementary Figure 6, and show a nonclassical redshift for the former and a blueshift for the latter, consistent with the fact that $\text{Re}\, d_\perp > 0$ for jellium metals and that $\text{Re}\, d_{\text{eff}} < 0$ for silver [4].

Higher-order localized surface plasmon resonances in plasmonic nanospheres. In the nonretarded regime, the l-dependent LSP resonances for deep subwavelength metal nanospheres follows from the implicit condition in Eq. (34). The ensuing LSP resonances for the first five multipoles—for a $r_s = 4$ jellium sphere with $R = 3\,\text{nm}$, embedded in a homogeneous medium with ϵ_{d}—are shown in Supplementary Figure 7

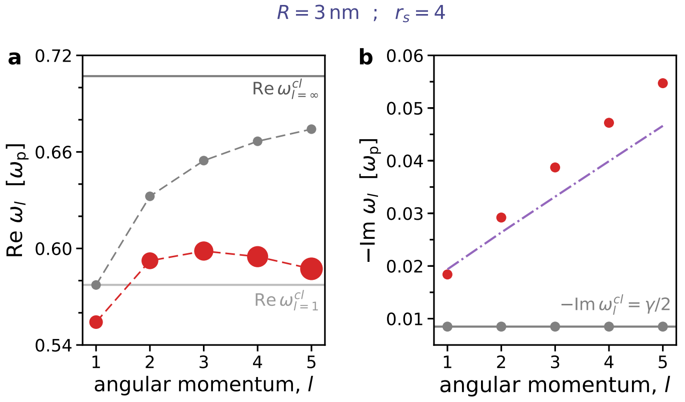

Supplementary Figure 7. Classical (gray circles) and quantum (red circles) higher-order LSP resonances in a R = 3 nm metal nanosphere (r_s = 4 jellium). In **a** the circle radius is proportional to the resonance width. Non-retarded LSP resonaces positions (**a**) and respective widths (**b**) as a function of the LSP angular momentum, l. The nanoparticle is assumed to be embedded in a host environment with dielectric constant ϵ_d = 1. The purple dashed-dot line indicates the *approximate* first-order correction predicted by Eqs. (7) of the main text [the deviation here is due to the pole-approximation performed owing to the explicit spectral dependence of the *d*-parameters—see discussion after the aforementioned Eqs. (7)].

SUPPLEMENTARY NOTE 6. NONCLASSICAL RESONANCE SHIFTS AND BROADENINGS

Figure 8 shows the relative nonclassical shifts and corresponding resonance widths for the two plasmonic structures considered in this work, a planar dielectric–metal interface and metal spheres or arbitrary radii. We emphasize that in Supplementary Figure 8 we employ the full solution—that is, including both retardation and the frequency dependence of the *d*-parameters—in order to obtain *quantitatively* accurate predictions. The usefulness of the *approximate* first-order spectral corrections given by Eqs. (7) of the

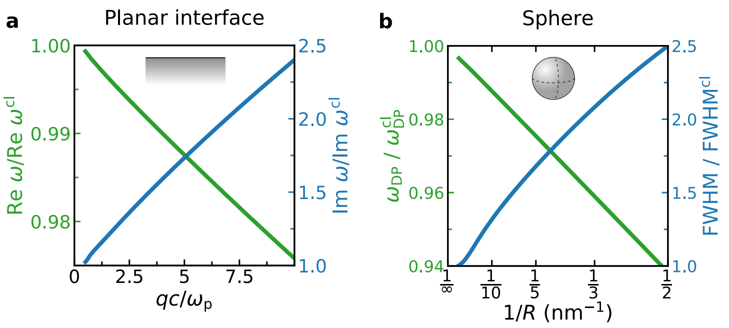

Supplementary Figure 8. Nonclassical plasmon resonance shifts and associated resonance widths for **a** an air–metal half-space; and **b** metal spheres (for the l = 1 dipole LSP). The plasmon-supporting material is assumed to be a jellium metal with r_s = 4.

main text hinges upon their ability to provide physical intuition and to elucidate in a *qualitative* fashion the scaling (with either q or R^{-1}) of the plasmonic spectral features.

SUPPLEMENTARY NOTE 7. QUANTUM CORRECTIONS TO THE OPTICAL LOCAL DENSITY OF STATES

Planar dielectric–metal interface

The (electric) local density of states (LDOS) experienced by an emitter with dipole moment $\boldsymbol{\mu}$ and located at a height h above a planar metal interface (defined by $z = 0$) is given by [27; 28]

$$\frac{\rho^{\mathrm{E}}_{\boldsymbol{\mu}}(\omega, h)}{\rho^{\mathrm{E}}_0(\omega)} = 1 + \frac{3}{4}\frac{\mu_x^2 + \mu_y^2}{|\boldsymbol{\mu}|^2} \operatorname{Re} \int_0^\infty \mathrm{d}u \, \frac{u}{\sqrt{1-u^2}} \left[r^{\mathrm{TE}} - (1-u^2) r^{\mathrm{TM}} \right] \mathrm{e}^{2\mathrm{i}k_{\mathrm{d}}h\sqrt{1-u^2}} + \frac{3}{2}\frac{\mu_z^2}{|\boldsymbol{\mu}|^2} \operatorname{Re} \int_0^\infty \mathrm{d}u \, \frac{u^3}{\sqrt{1-u^2}} \, r^{\mathrm{TM}} \, \mathrm{e}^{2\mathrm{i}k_{\mathrm{d}}h\sqrt{1-u^2}}, \tag{37}$$

where we have normalized the LDOS to its value in a homogeneous medium with relative permittivity ϵ_{d} (i.e., in the absence of the metal half-space). In the above, $r^{\mathrm{TM}} \equiv r^{\mathrm{TM}}(u, \omega)$ and $r^{\mathrm{TE}} \equiv r^{\mathrm{TE}}(u, \omega)$ are the reflection coefficients for a planar interface (cf. Supplementary Note SUPPLEMENTARY NOTE 3), and we have introduced the dimensionless variable $u \equiv q/k_{\mathrm{d}}$. Considering separately the cases of an ideal dipole emitter with perpendicular and parallel dipole moments (with the respect to the interface), we have

$$\frac{\rho^{\mathrm{E}}_{\perp}(\omega, h)}{\rho^{\mathrm{E}}_0(\omega)} = 1 + \frac{3}{2} \operatorname{Re} \int_0^\infty \mathrm{d}u \, \frac{u^3}{\sqrt{1-u^2}} \, r^{\mathrm{TM}} \, \mathrm{e}^{2\mathrm{i}k_{\mathrm{d}}h\sqrt{1-u^2}}, \tag{38a}$$

and

$$\frac{\rho^{\mathrm{E}}_{\parallel}(\omega, h)}{\rho^{\mathrm{E}}_0(\omega)} = 1 + \frac{3}{4} \operatorname{Re} \int_0^\infty \mathrm{d}u \, \frac{u}{\sqrt{1-u^2}} \left[r^{\mathrm{TE}} - (1-u^2) r^{\mathrm{TM}} \right] \mathrm{e}^{2\mathrm{i}k_{\mathrm{d}}h\sqrt{1-u^2}}, \tag{38b}$$

respectively. The corresponding orientation-averaged LDOS is then $\langle \rho^{\mathrm{E}} \rangle = \frac{1}{3}\rho^{\mathrm{E}}_{\perp} + \frac{2}{3}\rho^{\mathrm{E}}_{\parallel}$, which can be particularly useful when dealing with an ensemble of randomly-oriented dipoles (e.g., in dyes).

It should now be clear that the work in Supplementary Note SUPPLEMENTARY NOTE 3 pays off also for the calculation of the LDOS taking quantum mechanical effects into account, since one can simply employ the corresponding nonclassical reflection coefficients, derived in Supplementary Note SUPPLEMENTARY NOTE 3, in Eqs. (37) and (38) (with their classical equivalents naturally defining the classical LDOS). The results of such computations are summarized in Supplementary Figure 9.

Spherical metal sphere

The expression for the LDOS felt by an ideal electric dipole in the neighborhood of a spherical nanoparticle can be derived by following the same steps that lead to the LDOS in the planar case [29]. In particular, the LDOS for a radial ($\perp$) and for a tangent ($\parallel$) dipole near a metal sphere is given by[5] [29; 30]

$$\frac{\rho^{\mathrm{E}}_{\perp}(\omega, h)}{\rho^{\mathrm{E}}_0(\omega)} = 1 + \frac{3}{2}\frac{1}{y^2} \sum_{l=1}^{\infty} (2l+1) l(l+1) \operatorname{Re} \left\{ -a_l^{\mathrm{TM}} \left[h_l^{(1)}(y) \right]^2 \right\}, \tag{39a}$$

and

$$\frac{\rho^{\mathrm{E}}_{\parallel}(\omega, h)}{\rho^{\mathrm{E}}_0(\omega)} = 1 + \frac{3}{4}\frac{1}{y^2} \sum_{l=1}^{\infty} (2l+1) \operatorname{Re} \left\{ -a_l^{\mathrm{TM}} \left[\xi_l'(y) \right]^2 - a_l^{\mathrm{TE}} \left[\xi_l(y) \right]^2 \right\}, \tag{39b}$$

---

[5] We note that some authors use alternative definitions of the Mie coefficients, which may differ from ours by a minus sign. We have adopted a convention such that in the classical limit our expressions agree with those of Bohren and Huffman [25].

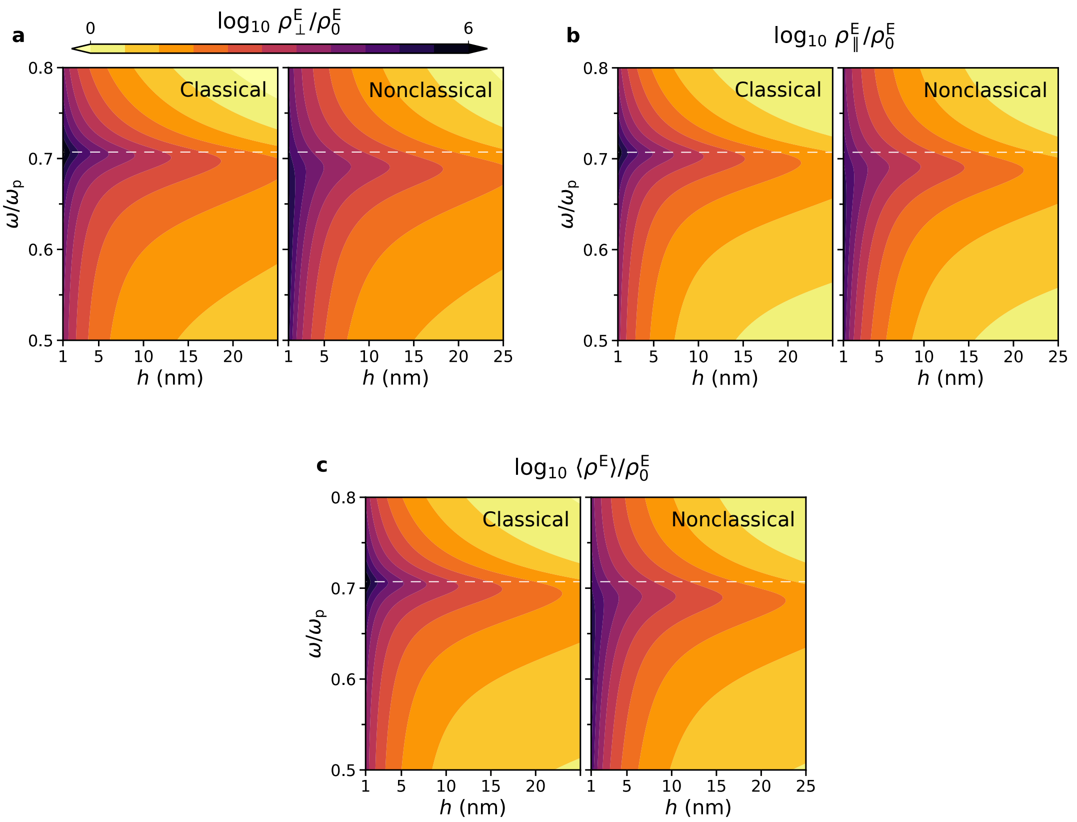

Supplementary Figure 9. Normalized LDOS (Purcell factor), $\rho^{\mathrm{E}}/\rho_0^{\mathrm{E}}$, perceived by an electric dipole at a distance h above a planar jellium surface ($r_s = 4$), with its dipole moment oriented along the **a** perpendicular ($\perp$) and **b** parallel ($\|$) directions with respect to the metal's surface. **c** Orientation-averaged dipole in the same configuration. The dielectric medium constituting the upper half-space is assumed to be air ($\epsilon_{\mathrm{d}} = 1$). The colorbar range is fixed in all panels to facilitate the comparison between the different cases. The horizontal lines mark the the nonretarded classical surface plasmon frequency, $\omega_{\mathrm{p}}/\sqrt{2}$.

where we have defined $y = k_{\mathrm{d}}(R+h) = x_{\mathrm{d}}(1+h/R)$ for the sake of clarity. Clearly, by comparing Eqs. (38) and (39) one realizes that the Mie coefficients in the latter play a similar role as the reflection coefficients in the former, and that the integration over in-plane momentum now appears as a sum over angular momenta instead, reflecting the (spherical) symmetry of the problem. Furthermore, and as before, the nonclassical LDOS possesses the same mathematical structure as its classical version. Hence, the quantum mechanical LDOS is immediately obtained upon substituting the classical Mie coefficients by their quantum mechanical counterparts. The outcome of Eqs. (39) is shown in Supplementary Figure 10.

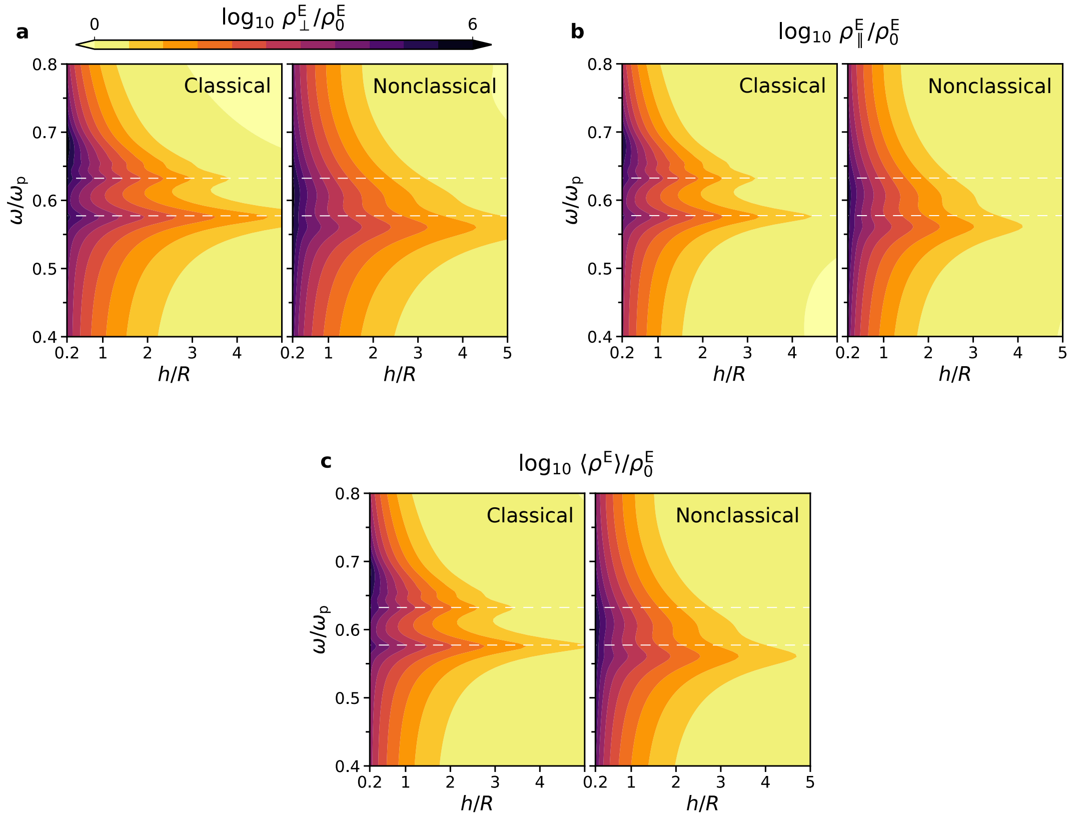

Supplementary Figure 10. Normalized LDOS, $\rho^{\mathrm{E}}/\rho_0^{\mathrm{E}}$, via Eqs. (39), experienced by an emitter in the vicinity of a metal sphere. The emitter is located at a distance h from the sphere's surface. The medium outside the sphere is assumed to be air ($\epsilon_{\mathrm{d}} = 1$) and the sphere is assumed to be constituted by a $r_s = 4$ jellium. **a** LDOS for a radial dipole ($\perp$); and **b** for a tangent ($\|$) dipole (with respect to the nanoparticle's surface). **c** Orientation-averaged dipole in the same configuration. The colorbar range is fixed in all panels to facilitate the comparison between the different cases. The horizontal dashed lines indicate the positions of the nonretarded classical LSP resonances for $l = 1, 2$.

SUPPLEMENTARY NOTE 8. ENHANCEMENT OF MULTIPOLAR DECAY RATES: INFLUENCE OF QUANTUM NONLOCAL EFFECTS

Atom-field Hamiltonian. In the context of quantum electrodynamics (QED) we consider atom-field interactions whose dynamics are governed by the following nonrelativistic Hamiltonian:

$$H = H_{\mathrm{a}} + H_{\mathrm{EM}} + H_{\mathrm{int}}, \tag{40a}$$

$$H_{\mathrm{a}} = \sum_n \left(\frac{p_n^2}{2m} - \frac{e^2}{4\pi\epsilon_0} \frac{1}{r_n} \right), \tag{40b}$$

$$H_{\mathrm{EM}} = \sum_{j=\{x,y,z\}} \int \mathrm{d}\mathbf{r} \int \mathrm{d}\omega\, \hbar\omega \left[f_j^\dagger(\mathbf{r},\omega) f_j(\mathbf{r},\omega) + \frac{1}{2} \right], \tag{40c}$$

$$H_{\mathrm{int}} = \sum_n \left[\frac{e}{2m} \left(\mathbf{p}_n \cdot \mathbf{A}(\mathbf{r}_n) + \mathbf{A}(\mathbf{r}_n) \cdot \mathbf{p}_n \right) + \frac{e}{2m} \mathbf{A}^2(\mathbf{r}_n) \right], \tag{40d}$$

where H_{a} is the atom Hamiltonian (or, more generically, of an emitter, e.g., a quantum dot, etc), H_{EM} is the Hamiltonian of the electromagnetic (EM) field, and H_{int} stands for the Hamiltonian governing the atom-field interaction. As written above, the matter Hamiltonian H_{a} is treated at the Hartree-Fock level (consisting of a kinetic part together with the Coulomb potential), that is, electron-electron interactions, spin-orbit coupling, etc, will be neglected in what follows. Naturally, in any case these may be readily incorporated here. Moreover, the Hamiltonian akin to the EM field is just the like the one of a typical

quantum harmonic oscillator, where the operator $f_j^\dagger(\mathbf{r},\omega)$ ($f_j(\mathbf{r},\omega)$) creates (annihilates) a field excitation at position $\mathbf{r}$ with frequency ω and oriented along the direction j. For the part of the Hamiltonian describing the interaction between the atom and the field, H_{int}, we take the so-called minimal-coupling interaction Hamiltonian[6]. Finally, $\mathbf{A}(\mathbf{r})$ is the vector potential associated with the EM field, from which both the electric and magnetic fields stem, in particular, $\mathbf{E}(\mathbf{r}) = -\frac{\partial}{\partial t}\mathbf{A}(\mathbf{r})$ and $\mathbf{H}(\mathbf{r}) = \mu_0^{-1}\nabla\times\mathbf{A}(\mathbf{r})$ [15; 27]. Notice that we have implicitly assumed that the scalar potential is identically zero (in our gauge). It should be noted that such vector and scalar potentials are simply useful mathematical entities/abstractions and, unlike $\mathbf{E}(\mathbf{r})$ and $\mathbf{B}(\mathbf{r})$, do not represent *per se* real physical quantities.

Below, we will be dealing with (linear) dispersive and dissipative media and therefore the common canonical quantization based on modal expansion is unsuitable. Ergo, we employ instead the formalism of macroscopic quantum electrodynamics [31; 32].

Dyadic Green's functions and macroscopic QED. From Maxwell's equations, the following wave equation is immediately apparent (assuming linear, isotropic, and nonmagnetic media) [15]:

$$\nabla\times\nabla\times\mathbf{E}(\mathbf{r},\omega) - \frac{\omega^2}{c^2}\epsilon(\mathbf{r},\omega)\mathbf{E}(\mathbf{r},\omega) = \mathrm{i}\omega\mu_0\mathbf{j}(\mathbf{r},\omega). \tag{41}$$

The solution of Eq. (41) can then be written in terms of the associated dyadic Green's function, in particular

$$\mathbf{E}(\mathbf{r},\omega) = \mathrm{i}\omega\mu_0\overleftrightarrow{\mathbf{G}}(\mathbf{r},\mathbf{r}';\omega)\cdot\mathbf{j}(\mathbf{r},\omega), \tag{42}$$

where the Green's dyadic satisfies

$$\nabla\times\nabla\times\overleftrightarrow{\mathbf{G}}(\mathbf{r},\mathbf{r}';\omega) - \frac{\omega^2}{c^2}\epsilon(\mathbf{r},\omega)\overleftrightarrow{\mathbf{G}}(\mathbf{r},\mathbf{r}';\omega) = \boldsymbol{\delta}(\mathbf{r}-\mathbf{r}'). \tag{43}$$

Furthermore, we note the following useful properties of the Green's function [31]:

$$\overleftrightarrow{\mathbf{G}}^*(\mathbf{r},\mathbf{r}';\omega) = \overleftrightarrow{\mathbf{G}}(\mathbf{r},\mathbf{r}';-\omega^*), \tag{44a}$$

$$\overleftrightarrow{\mathbf{G}}(\mathbf{r}',\mathbf{r};\omega) = \overleftrightarrow{\mathbf{G}}^T(\mathbf{r},\mathbf{r}';\omega), \tag{44b}$$

$$\frac{\omega^2}{c^2}\int \mathrm{d}\mathbf{x}\,\mathrm{Im}\{\epsilon(\mathbf{x},\omega)\}\overleftrightarrow{\mathbf{G}}(\mathbf{r},\mathbf{x};\omega)\cdot\overleftrightarrow{\mathbf{G}}^\dagger(\mathbf{r}',\mathbf{x};\omega) = \mathrm{Im}\,\overleftrightarrow{\mathbf{G}}(\mathbf{r},\mathbf{r}';\omega), \tag{44c}$$

where the first and second identities reflect, respectively, the analytical properties of the Green's dyadic function and reciprocity.

Furthermore, the current $\mathbf{j}(\mathbf{r},\omega)$—representing a dynamic variable of the system composed by the EM field and the medium—may be written as [31; 32]

$$j_i(\mathbf{r},\omega) = \omega\sqrt{\frac{\hbar\epsilon_0}{\pi}\,\mathrm{Im}\,\epsilon(\mathbf{r},\omega)}f_i(\mathbf{r},\omega), \tag{45}$$

where the bosonic field operators obey the commutation relations:

$$\left[f_i(\mathbf{r},\omega), f_j^\dagger(\mathbf{r}',\omega')\right] = \delta_{i,j}\delta(\mathbf{r}-\mathbf{r}')\delta(\omega-\omega'), \tag{46a}$$

$$\left[f_i(\mathbf{r},\omega), f_j(\mathbf{r}',\omega')\right] = 0. \tag{46b}$$

Hence, using Eqs. (42) and (45), one obtains

$$\mathbf{E}(\mathbf{r},\omega) = \mathrm{i}\frac{\omega^2}{c^2}\sqrt{\frac{\hbar}{\pi\epsilon_0}}\int \mathrm{d}\mathbf{r}'\,\sqrt{\mathrm{Im}\,\epsilon(\mathbf{r}',\omega)}\overleftrightarrow{\mathbf{G}}(\mathbf{r},\mathbf{r}';\omega)\cdot\mathbf{f}(\mathbf{r}',\omega), \tag{47}$$

---

[6] If spin-orbit coupling is considered, then H_{int} would also have an extra term, $\frac{e\hbar}{2m}\boldsymbol{\sigma}\cdot\mathbf{B}(\mathbf{r})$ (where $\boldsymbol{\sigma}$ denotes the Pauli matrices), responsible for the interaction of of the particle's spin with the magnetic field of the EM radiation.

so that, while realizing that in our gauge $\mathbf{E}(\mathbf{r},\omega) = \mathrm{i}\omega\mathbf{A}(\mathbf{r},\omega)$, we find

$$\mathbf{A}(\mathbf{r},\omega) = \frac{\omega}{c^2}\sqrt{\frac{\hbar}{\pi\epsilon_0}}\int \mathrm{d}\mathbf{r}'\,\sqrt{\mathrm{Im}\,\epsilon(\mathbf{r}',\omega)}\overleftrightarrow{\mathbf{G}}(\mathbf{r},\mathbf{r}';\omega)\cdot\mathbf{f}(\mathbf{r}',\omega). \tag{48}$$

From here, and noting that $\mathbf{A}(\mathbf{r}) = \int \mathrm{d}\omega\,\mathbf{A}(\mathbf{r},\omega) + \mathrm{H.c.}$, we now write the components of the vector potential operator featuring in the interaction Hamiltonian, Eq. (40d), as

$$A_i(\mathbf{r}) = \sqrt{\frac{\hbar}{\pi\epsilon_0}}\int \mathrm{d}\omega\,\frac{\omega}{c^2}\int \mathrm{d}\mathbf{r}'\,\sqrt{\mathrm{Im}\,\epsilon(\mathbf{r}',\omega)}G_{ij}(\mathbf{r},\mathbf{r}';\omega)f_j(\mathbf{r}',\omega) + \mathrm{H.c.}, \tag{49}$$

where summation over repeated indices is implicitly assumed (Einstein's summation notation).

Fermi's Golden Rule and first-order processes. In what follows we consider electronic first-order processes alone. This means, for instance, that we neglect the second term of the minimal-coupling Hamiltonian in Eq. (40d) [i.e., the term proportional to $\mathbf{A}^2 \equiv \mathbf{A}\cdot\mathbf{A}$]. Moreover, for definiteness, we assume that the emitter is a Hydrogen atom. Hence, the atom-field interaction Hamiltonian then becomes

$$H_{\mathrm{int}} = \frac{e}{2m}\left[\mathbf{p}\cdot\mathbf{A}(\mathbf{r}) + \mathbf{A}(\mathbf{r})\cdot\mathbf{p}\right]. \tag{50}$$

We want to determine the transition rate between an initial (excited) state $|i\rangle = |e,0\rangle \equiv |e\rangle\otimes|0\rangle$ and a final (not necessarily the lowest) state $|f\rangle = |g, 1_{\mathbf{x}j\omega_0}\rangle = f_j^\dagger(\mathbf{x},\omega_0)\,|g,0\rangle$. The transition rate for such a first-order process calculated within first-order perturbation theory follows from Fermi's Golden Rule

$$\Gamma_{i\to f} = \frac{2\pi}{\hbar^2}\,|\langle f|\,H_{\mathrm{int}}\,|i\rangle|^2\,\delta(\omega_i - \omega_f - \omega). \tag{51}$$

The matrix element entering in Eq. (51) reads[7]

$$\begin{aligned}
\langle f|\,H_{\mathrm{int}}\,|i\rangle &= \frac{e}{m}\sum_k \langle g, 1_{\mathbf{x}k\omega_0}|\,\mathbf{A}(\mathbf{r})\cdot\mathbf{p}\,|e,0\rangle \\
&= \frac{e}{m}\sum_k \langle g,0|\,f_k(\mathbf{x},\omega_0)\mathbf{A}(\mathbf{r})\cdot\mathbf{p}\,|e,0\rangle \\
&= \frac{e}{m}\sqrt{\frac{\hbar}{\pi\epsilon_0}}\sum_k \langle g,0|\,f_k(\mathbf{x},\omega_0)\int \mathrm{d}\omega\,\frac{\omega}{c^2}\int \mathrm{d}\mathbf{r}'\;\sqrt{\mathrm{Im}\,\epsilon(\mathbf{r}',\omega)}G_{ij}(\mathbf{r},\mathbf{r}';\omega)f_j(\mathbf{r}',\omega)p_i\,|e,0\rangle \\
&+ \frac{e}{m}\sqrt{\frac{\hbar}{\pi\epsilon_0}}\sum_k \langle g,0|\,f_k(\mathbf{x},\omega_0)\int \mathrm{d}\omega\,\frac{\omega}{c^2}\int \mathrm{d}\mathbf{r}'\;\sqrt{\mathrm{Im}\,\epsilon(\mathbf{r}',\omega)}f_m^\dagger(\mathbf{r}',\omega)G_{mn}^\dagger(\mathbf{r},\mathbf{r}';\omega)p_n\,|e,0\rangle \\
&= \frac{e}{m}\sqrt{\frac{\hbar}{\pi\epsilon_0}}\frac{\omega_0}{c^2}\sum_k \langle g,0|\;\sqrt{\mathrm{Im}\,\epsilon(\mathbf{x},\omega_0)}G_{kn}^\dagger(\mathbf{r},\mathbf{x};\omega_0)p_n\,|e,0\rangle\,.
\end{aligned} \tag{52}$$

where the commutation relations of the bosonic field operators have been used. Carrying out a similar calculation for $\langle i|\,H_{\mathrm{int}}\,|f\rangle$, and making use of the identity in Eq. (44c), one may write (taking the wave-functions as real)

$$\Gamma = \frac{2\pi}{\hbar^2}\frac{e^2\hbar}{\pi\epsilon_0 m^2c^2}\int \mathrm{d}\mathbf{r}\int \mathrm{d}\mathbf{r}'\,\psi_e(\mathbf{r}')\psi_e(\mathbf{r})\,\mathrm{Im}\,G_{ij}(\mathbf{r},\mathbf{r}';\omega_0)(p_i\psi_g(\mathbf{r}))(p_j^*\psi_g(\mathbf{r}')). \tag{53}$$

Now, the electromagnetic Green's function admits an analytical expression of the form (for $z > 0$)

$$\overleftrightarrow{\mathbf{G}}(\mathbf{r},\mathbf{r}';\omega_0) = \frac{\mathrm{i}}{8\pi^2}\int \mathrm{d}\mathbf{q}\left[\overleftrightarrow{\mathbf{M}}_s + \overleftrightarrow{\mathbf{M}}_p\right]\mathrm{e}^{\mathrm{i}\mathbf{q}\cdot\boldsymbol{\rho}+\mathrm{i}k_z z}\mathrm{e}^{-\mathrm{i}\mathbf{q}\cdot\boldsymbol{\rho}'+\mathrm{i}k_z z'}. \tag{54}$$

---

[7] Neglecting the contribution from $\nabla\cdot\mathbf{A}$ which is approximately zero away from the metal (where $\nabla\cdot\mathbf{A} = 0$).

Since deviations of the LDOS from classicality owing to quantum surface corrections incur at relatively short emitter–metal distances, i.e., at relatively large wavevectors, we here assume the nonretarded limit, i.e., $q \gg k_0$ (except for the reflection coefficient, which is still assumed to be the retarded version in order to obtain the plasmon pole in accurately), such that $k_z \to \mathrm{i}q$ and $\|\overleftrightarrow{\mathbf{M}}_s\| \to 0$ as $c \to \infty$:

$$\overleftrightarrow{\mathbf{G}}(\mathbf{r},\mathbf{r}';\omega_0) \overset{c\to\infty}{\simeq} \frac{\mathrm{i}}{8\pi^2}\int \mathrm{d}\mathbf{q}\,\overleftrightarrow{\mathbf{M}}_p\Big|_{c\to\infty} \mathrm{e}^{\mathrm{i}\mathbf{q}\cdot\boldsymbol{\rho}-qz}\mathrm{e}^{-\mathrm{i}\mathbf{q}\cdot\boldsymbol{\rho}'-qz'}, \tag{55}$$

where

$$\overleftrightarrow{\mathbf{M}}_p\Big|_{c\to\infty} = -\frac{qc^2}{\omega_0^2}r^{\mathrm{TM}}\begin{pmatrix}\mathrm{i} & 0 & 1\\ 0 & 0 & 0\\ -1 & 0 & \mathrm{i}\end{pmatrix} = -\frac{qc^2}{\omega_0^2}r^{\mathrm{TM}}\begin{pmatrix}\mathrm{i}\\ 0\\ -1\end{pmatrix}\otimes\begin{pmatrix}1 & 0 & -\mathrm{i}\end{pmatrix} \equiv -2\mathrm{i}\frac{qc^2}{\omega_0^2}r^{\mathrm{TM}}\hat{\boldsymbol{\epsilon}}_{\hat{\mathbf{q}}}\otimes\hat{\boldsymbol{\epsilon}}^*_{\hat{\mathbf{q}}}, \tag{56}$$

with $\hat{\boldsymbol{\epsilon}}_{\hat{\mathbf{q}}}$ denoting the polarization vector defined via $\hat{\boldsymbol{\epsilon}}_{\hat{\mathbf{q}}} = (\hat{\mathbf{q}} + \mathrm{i}\hat{\mathbf{z}})/\sqrt{2}$. The transition rate in Eq. (53) then reduces to

$$\Gamma = \frac{e^2}{2\pi^2\epsilon_0\hbar m^2\omega_0^2}\int_0^{2\pi}\mathrm{d}\varphi\int_0^{\infty}\mathrm{d}q\,q^2\left|\langle e|\,(\hat{\boldsymbol{\epsilon}}_{\hat{\mathbf{q}}}\cdot\mathbf{p})\mathrm{e}^{\mathrm{i}\mathbf{q}\cdot\boldsymbol{\rho}-qz}\,|g\rangle\right|^2\mathrm{Im}\,r^{\mathrm{TM}}. \tag{57}$$

It is useful to rewrite Eq. (57) as

$$\Gamma = \frac{e^2}{2\pi^2\epsilon_0\hbar m^2\omega_0^2}\int_0^{2\pi}\mathrm{d}\varphi\int_0^{\infty}\mathrm{d}q\,q^2\left|\langle e|\,(\hat{\boldsymbol{\epsilon}}_{\hat{\mathbf{q}}}\cdot\mathbf{p})\mathrm{e}^{\mathrm{i}\mathbf{q}\cdot\boldsymbol{\rho}-q(z-z_0)}\,|g\rangle\right|^2\mathrm{Im}\,r^{\mathrm{TM}}, \tag{58}$$

which allows the evaluation of the matrix element with the emitter at the origin.

For simplicity, in what follows we restrict ourselves to electric multipolar transitions whose initial and final states posses $m_f = m_i = 0$. Then, we may choose $\mathbf{q}$ along the x-direction, and, writing the momentum operator as $\mathbf{p} = -\mathrm{i}\hbar\nabla$, we obtain

$$\Gamma = \frac{4\alpha\hbar^2\omega_0}{m^2c^2}\int_0^{\infty}\mathrm{d}u\,u^2\mathrm{e}^{-2uk_0z_0}\left|\langle e|\,\mathrm{e}^{\mathrm{i}uk_0x-uk_0(z-z_0)}\hat{\boldsymbol{\epsilon}}_{\hat{\mathbf{q}}}\cdot\nabla\,|g\rangle\right|^2\mathrm{Im}\,r^{\mathrm{TM}}, \tag{59}$$

where we have introduced the dimensionless variable $u \equiv q/k_0$. Since for an arbitrary nth electric transition, En, the matrix element in Eq. (59) is dominated by only one of the terms coming from the expansion of the exponential,[8] we get

$$\left|\langle e|\,\mathrm{e}^{\mathrm{i}k_0ux-k_0u(z-z_0)}\hat{\boldsymbol{\epsilon}}_{\hat{\mathbf{q}}}\cdot\nabla\,|g\rangle\right|^2 \propto u^{2(n-1)}. \tag{60}$$

Finally, this leads to the following expression of the transition rate of a given hydrogenic En transition:

$$\Gamma_{\mathrm{E}n} = 2\alpha^3\omega_0\left[\frac{(k_0a_{\mathrm{B}})^{n-1}}{(n-1)!}\right]^2\Xi^{l_e,l_g}_{n_e,n_g}\int_0^{\infty}\mathrm{d}u\,u^{2n}\mathrm{e}^{-2uk_0z_0}\,\mathrm{Im}\,r^{\mathrm{TM}}, \tag{61}$$

where a_{B} denotes the Bohr radius and where the quantity Ξ originates from the matrix element,

$$\begin{aligned}\Xi^{l_e,l_g}_{n_e,n_g} \equiv \Bigg|\int_0^{2\pi}\mathrm{d}\phi\int_0^{\pi}\mathrm{d}\theta\,\sin\theta\int_0^{\infty}\mathrm{d}\bar{r}\,\bar{r}^2\,\Psi_e(\bar{r},\theta,\phi)\,(\mathrm{i}\bar{r}\sin\theta\cos\phi-\bar{r}\cos\theta)^{n-1}\\ \times(\sin\theta\cos\phi+\mathrm{i}\cos\theta)\frac{\partial}{\partial\bar{r}}\Psi_g(\bar{r},\theta,\phi)\Bigg|^2,\end{aligned} \tag{62}$$

with $\bar{r} \equiv r/a_{\mathrm{B}}$ (note that here the wavefunctions Ψ are dimensionless).

[8] In particular, the dominating term arising from the expansion of the exponential is the one corresponding to change in the angular momentum of the atom by an integer n, i.e., $|l_f - l_i| = n$ (see also the Supplementary Material of Ref. 33). This approximation essentially amounts to a point-emitter assumption, with an associated $O[(qa_{\mathrm{B}})^{2(n-1)}]$ accuracy. For the dipole transition, inclusion of terms beyond the $u^{2(n-1)}$ term produce so-called non-dipolar corrections [34]; for a general En transition, their inclusion produce non-n-polar corrections. These corrections can be substantial in "large" emitters (e.g., quantum dots or molecules).

SUPPLEMENTARY NOTE 9. BROADBAND PHENOMENA: NONCLASSICAL EFFECTS

Prelude: extrapolation of *d*-parameters beyond the plasma frequency using sum-rules

To extrapolate the argument-range of the $d_\perp$ parameter beyond ω_p, we perform a multi-Lorentzian fitting of previous data [3] that cover $d_\perp$ across $0 < \omega < \omega_\mathrm{p}$. By enforcing sum-rules and appropriate asymptotics [35; 36], we anticipate that the fit maintains a reasonable validity in the extrapolating domain $\omega > \omega_\mathrm{p}$.

Fitting scheme

We seek a fit of the jellium data of Ref. 3 to a sum of Lorentzians:

$$d_\perp^{\mathrm{fit}}(\omega) = \sum_{n=1}^{N} \frac{f_n}{\omega(\omega + \mathrm{i}\gamma_n) - \omega_n^2}, \tag{63}$$

with $f_n \in \mathbb{C}$, $\gamma_n \in \mathbb{R}$, and $\omega_n \in \mathbb{R}$. In addition, we impose the following basic requirements on each of the oscillator parameters:

Amplitudes f_n. To strictly ensure the large-ω behavior $\operatorname{Im} d_\perp(\omega \gg \omega_\mathrm{p}) \sim \omega^{-3}$, we explicitly enforce that $\sum_{n=1}^{N} \operatorname{Im} f_n = 0$ by setting $\operatorname{Im} f_N = -\sum_{n=1}^{N-1} \operatorname{Im} f_n$.

Resonance widths γ_n. To ensure causality, γ_n is restricted to positive real values, i.e., $\gamma_n > 0$. This is enforced via a penalty term.

Resonance frequencies ω_n. In the expectation that all resonant behavior occurs below ω_p, we restrict $\omega_n^2 \in [0, \omega_\mathrm{p}^2]$. This is enforced via penalty terms. To strictly ensure that $\operatorname{Im} d_\perp(\omega \to 0) = 0$, we explicitly enforce that $\sum_{n=1}^{N} \operatorname{Im} f_n/\omega_n^2 = 0$ by setting $\omega_N^2 = -(\operatorname{Im} f_N)/(\sum_{n=1}^{N-1} \operatorname{Im} f_n/\omega_n^2)$.

In addition, we enforce the following sum-rules[9] [35] via penalty terms:

$$\int_0^\infty \operatorname{Re} d_\perp(\omega)\, \mathrm{d}\omega = 0, \tag{64}$$

$$\int_0^\infty \frac{\operatorname{Im} d_\perp(\omega)}{\omega}\, \mathrm{d}\omega = \frac{\pi}{2} \operatorname{Re} d_\perp(0). \tag{65}$$

Finally, we enforce that $\operatorname{Im} d_\perp(\omega > \omega_\mathrm{p}) < 0$ to ensure that power is absorbed rather than gained at these frequencies [the opposite is the case for frequencies below ω_p since the surface power absorption is proportional to $\varepsilon(\omega) d_\perp(\omega)$].

The fitting itself is performed by minimizing the absolute square of residuals plus the penalty-terms noted above. The minimization itself is performed by the Julia package `Optim.jl` [37], using the Nealder–Mead algorithm.

Results

We've carried out this fitting procedure for $r_s = 4$. We obtain a reasonable fit and small relative deviation from the sum-rules with five oscillator terms. The results are summarized in Supplementary Table 1.[10]

---

[9] There are additional sum-rules, that we nevertheless do not enforce, e.g., $\int_0^\infty \omega \operatorname{Im} d_\perp(\omega)\, \mathrm{d}\omega = -\eta$ where η is a large-frequency asymptotic scaling-parameter of $\operatorname{Re} d_\perp$ defined by $\operatorname{Re} d_\perp(\omega \to \infty) = 2\pi^{-1}\eta\omega^{-2}$.

[10] The Fermi momentum k_F relates with the dimensionless Wigner–Seitz radius as $k_\mathrm{F} r_s a_\mathrm{B} = \sqrt[3]{9\pi/4}$ (with Bohr radius $a_\mathrm{B} \approx 0.529$ Å)

n	Re $f_n k_F/\omega_p^2$	Im $f_n k_F/\omega_p^2$	ω_n^2/ω_p^2	γ_n/ω_p	$\lvert f_n\rvert k_F/\omega_p^2$
1	−0.40133	−0.0225044	0.664958	0.107995	0.401961
2	1.13998	0.0855696	0.909115	0.264916	1.143190
3	−0.91171	−0.00176816	0.890639	0.206974	0.911713
4	−0.0978378	0.00235707	0.439866	0.287170	0.0978662
5	0.0453377	−0.0636541	1.0	0.0120255	0.0781496

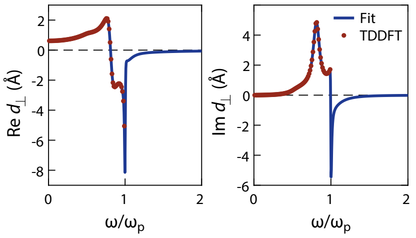

Supplementary Table 1. Multi-Lorentzian fitting coefficients for $r_s = 4$. Passes sum-rule (65) (relative error of 0.0%); nearly passes sum-rule (64) (relative error of 3.1%, relative to weight below ω_p); and the mean square error relative to (absolute mean square of) the original dataset is 0.03%. Figures visually illustrate the high fit quality.

Energy transfer between two dipoles

The total energy transfer rate from a donor (D) to an acceptor (A), characterized by a dipole moment $\boldsymbol{\mu}_D$ and $\boldsymbol{\mu}_A$, respectively, follows from [27; 38–40]

$$\Gamma_{ET} = \int w_{ET}(\mathbf{r}_D, \mathbf{r}_A, \omega) f_D^{em}(\omega) f_A^{abs}(\omega)\, d\omega, \tag{66a}$$

where the kernel $w_{ET}(\mathbf{r}_D, \mathbf{r}_A, \omega)$ is the energy transfer amplitude, given by

$$w_{ET}(\mathbf{r}_D, \mathbf{r}_A, \omega) = \frac{2\pi}{\hbar^2}\left(\frac{\omega^2}{\epsilon_0 c^2}\right)^2 \left|\boldsymbol{\mu}_A^* \cdot \overleftrightarrow{\mathbf{G}}(\mathbf{r}_D, \mathbf{r}_A; \omega) \cdot \boldsymbol{\mu}_D\right|^2. \tag{66b}$$

Notice that all the information about the nanophotonic environment is embodied in the Green's dyadic. Therefore, if the Green's function is either known or can be calculated for the system under consideration, then the rate of energy transfer can be determined by evaluating Eq. (66). For the half-plane and sphere plasmonic structures, the nonclassical energy transfer rates are then straightforwardly obtained by simply substituting the classical scattering coefficients by their corresponding nonclassical ones (namely, Eqs. (9) and (11) for the planar interface, and Eqs. (30)) and (31) for the sphere).

At this point it is also instructive to note that, contrary to the computation of the LDOS where the (imaginary part of the) Green's function is evaluated at the same point ($\mathbf{r} = \mathbf{r}' = \mathbf{r}_0$), the rate of energy transfer depends on the (absolute square of the) Green's function taken simultaneously at the positions of the donor and of the acceptor. Furthermore, for the calculation of the total (broadband) energy transfer rate one has to perform an integration over frequency—albeit typically restricted to the overlap between the emission spectrum of the donor, $f_D^{em}(\omega)$, and the absorption spectrum of the acceptor, $f_A^{abs}(\omega)$.

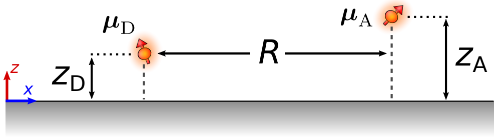

Supplementary Figure 11. Illustration of two dipoles in the vicinity of a planar metal surface. The dipole–surface separation is given by $z_{D(A)}$ for the donor (acceptor), and its dipole moment is characterized by $\mu_{D(A)}$. Without loss of generality, we choose our coordinate system so that $R = |x_D - x_A|$.

We now consider two electric point-dipoles (e.g., each a generic two-level system, or dye molecules, or quantum dots, etc) above a planar metal surface, as illustrated in Supplementary Figure 11. Therefore, according to Eqs. (66) the enhancement (with respect to the energy transfer rate in same medium but without the interface) of the energy transfer rate between the donor and the acceptor can be calculated as

$$\frac{\Gamma_{ET}}{\Gamma_{ET}^0} = \frac{\int_0^\infty d\omega\, \omega^4 \left|\hat{\mathbf{n}}_A \cdot \left[\overleftrightarrow{\mathbf{G}}_0(\mathbf{r}_D, \mathbf{r}_A; \omega) + \overleftrightarrow{\mathbf{G}}_{ref}(\mathbf{r}_D, \mathbf{r}_A; \omega)\right] \cdot \hat{\mathbf{n}}_D\right|^2 f_D^{em}(\omega) f_A^{abs}(\omega)}{\int_0^\infty d\omega\, \omega^4 \left|\hat{\mathbf{n}}_A \cdot \overleftrightarrow{\mathbf{G}}_0(\mathbf{r}_D, \mathbf{r}_A; \omega) \cdot \hat{\mathbf{n}}_D\right|^2 f_D^{em}(\omega) f_A^{abs}(\omega)}, \tag{67}$$

where $\overleftrightarrow{\mathbf{G}}_0(\mathbf{r}_\mathrm{D},\mathbf{r}_\mathrm{A};\omega)$ and $\overleftrightarrow{\mathbf{G}}_\mathrm{ref}(\mathbf{r}_\mathrm{D},\mathbf{r}_\mathrm{A};\omega)$ are the Green's dyadics associated with the homogeneous medium and with the reflected part owing to the metal surface, correspondingly. Here, $\hat{\mathbf{n}}_\mathrm{A}$ and $\hat{\mathbf{n}}_\mathrm{D}$ are unit vectors denoting, respectively, the orientation of the acceptor and donor dipole moments.

Example I: two vertical dipoles. For $\hat{\mathbf{n}}_\mathrm{D} = \hat{\mathbf{n}}_\mathrm{A} = \hat{\mathbf{z}}$, the relevant element of the $\overleftrightarrow{\mathbf{G}}_\mathrm{ref}(\mathbf{r}_\mathrm{D},\mathbf{r}_\mathrm{A};\omega)$ dyadic is given by

$$\left[\overleftrightarrow{\mathbf{G}}_\mathrm{ref}(\mathbf{r}_\mathrm{D},\mathbf{r}_\mathrm{A};\omega)\right]_{zz} = \frac{\mathrm{i}}{4\pi k_0^2}\int_0^\infty \frac{q^3}{k_z} J_0(qR) r^\mathrm{TM} \mathrm{e}^{\mathrm{i}k_z(z_D+z_A)}\,\mathrm{d}q, \tag{68}$$

with $k_z \equiv (k_0^2 - q^2)^{1/2}$, and where J_n is the nth order Bessel function of the first kind. This is the example highlighted in the main text.

Example II: two horizontal dipoles. For $\hat{\mathbf{n}}_\mathrm{D} = \hat{\mathbf{n}}_\mathrm{A} = \hat{\mathbf{x}}$, the relevant entry of the $\overleftrightarrow{\mathbf{G}}_\mathrm{ref}(\mathbf{r}_\mathrm{D},\mathbf{r}_\mathrm{A};\omega)$ dyadic is given by

$$\begin{aligned}\left[\overleftrightarrow{\mathbf{G}}_\mathrm{ref}(\mathbf{r}_\mathrm{D},\mathbf{r}_\mathrm{A};\omega)\right]_{xx} = {} & \frac{\mathrm{i}}{8\pi}\int_0^\infty \frac{q}{k_z}\left[J_0(qR) + J_2(qR)\right] r^\mathrm{TE} \mathrm{e}^{\mathrm{i}k_z(z_D+z_A)}\,\mathrm{d}q \\ & - \frac{\mathrm{i}}{8\pi k_0^2}\int_0^\infty q k_z \left[J_0(qR) - J_2(qR)\right] r^\mathrm{TM} \mathrm{e}^{\mathrm{i}k_z(z_D+z_A)}\,\mathrm{d}q.\end{aligned} \tag{69}$$

Second-order processes: two-photon emission

The theory of two-photon emission in arbitrary macroscopic media was derived in Ref. 33 and 41. Here, we summarize the main results, and defer to these references for additional details. From the standpoint of macroscopic quantum electrodynamics, two-photon emission can be represented as a transition from an initial state $|i\rangle \equiv |e,0\rangle$ (an excited matter state e and zero photons) to a final state $|f\rangle \equiv |g,\{\mathbf{r}\omega k,\mathbf{r}'\omega' k'\}\rangle$ (a lower-energy matter state g and two photons at positions $\mathbf{r}$ and $\mathbf{r}'$, frequencies ω and ω', and orientations k and k'). The energy difference between matter states is $\omega_0 \equiv \omega_e - \omega_g$. The rate of two-photon emission is then governed by Fermi's Golden rule at second-order in quantum electrodynamics, which takes the form

$$\Gamma_\mathrm{TPE} = \frac{2\pi}{\hbar^2}\frac{1}{2}\int \mathrm{d}\mathbf{r}\,\mathrm{d}\mathbf{r}' \int \mathrm{d}\omega\,\mathrm{d}\omega' \sum_{kk'}\left|\sum_{i_1} \frac{\langle g,\{\mathbf{r}\omega k,\mathbf{r}'\omega' k'\}|V|i_1\rangle\langle i_1|V|e,0\rangle}{E_e - E_{i_1} + \mathrm{i}0^+}\right|^2 \delta(\omega_0 - \omega - \omega'), \tag{70}$$

where $|i_1\rangle$ are intermediate states containing both the atom and field degrees of freedom. The sum is understood to be a sum over discrete degrees of freedom and an integral over continuous ones. The factor of $1/2$ comes from the fact that when we sum over all $\{\mathbf{r}\omega k,\mathbf{r}'\omega' k'\}$ pairs, each pair of excitations appears twice. As we will calculate two-photon emission within the dipole approximation, $V = -\mathbf{d}\cdot\mathbf{E}$ is the dipole Hamiltonian. After a series of manipulations, it is found that the second-order differential decay rate $\mathrm{d}\Gamma_\mathrm{TPE}/\mathrm{d}\omega$—i.e., the decay rate per unit frequency, defined such that $\Gamma_\mathrm{TPE} = \int_0^{\omega_0}\mathrm{d}\omega\,(\mathrm{d}\Gamma_\mathrm{TPE}/\mathrm{d}\omega)$—is given by:

$$\frac{\mathrm{d}\Gamma_\mathrm{TPE}}{\mathrm{d}\omega} = \frac{144\pi\alpha^2}{9c^2}\omega^2(\omega_0-\omega)^2\sum_{ijrs}\mathrm{Im}\,G_{ri}(\mathbf{r},\mathbf{r},\omega)\,\mathrm{Im}\,G_{sj}(\mathbf{r},\mathbf{r},\omega_0-\omega)T_{ij}(\omega)T^*_{rs}(\omega), \tag{71}$$

where T_{ij} is an atomic structure factor given by

$$T_{ij}(\omega) \equiv \sum_n \frac{x_j^{gn}x_i^{ne}}{\omega_e - \omega_n - \omega} + \frac{x_i^{gn}x_j^{ne}}{\omega_e - \omega_n - (\omega_0 - \omega)} = T_{ji}(\omega_0 - \omega), \tag{72}$$

where $x_j^{gn} \equiv \langle g|x_j|n\rangle$ is the transition matrix-element between matter states $|g\rangle$ and $|n\rangle$ along the jth Cartesian direction. We note that this derivation makes no assumption about the Green's function $\overleftrightarrow{\mathbf{G}}$ (with

elements G_{ij}), and therefore may be used to describe nonclassical corrections to two-photon emission via d-parameters.

The expression for the two-photon spectrum is quite general. Now, we specialize it both for the photonic structure and the emitter. For the photon, we consider a planar system (with in-plane translational symmetry). For such a translationally invariant system (and taking a nonretarded, high-Purcell enhancement approximation; both well-realized in our considerations), one has $\operatorname{Im} G_{ij}(\mathbf{r},\mathbf{r},\omega) = \frac{\omega}{6\pi c} F_{\mathrm{p}}(\mathbf{r},\omega) D_{ij}$, where $D \equiv \mathrm{diag}(1/2, 1/2, 1)$ and $F_{\mathrm{p}}(\omega) \equiv \rho_{\perp}^{\mathrm{E}}(\mathbf{r},\omega)/\rho_0^{\mathrm{E}}(\omega)$ is the Purcell factor for one-photon emission for a perpendicularly-oriented dipole (frequency ω and position $\mathbf{r}$) near this material (and $\rho_0^{\mathrm{E}}(\omega) \equiv \omega^2/\pi^2 c^3$). For the matter, we consider a hydrogenic atom undergoing a transition between s-states. Under these assumptions, the two-photon differential decay rate is given by

$$\frac{\mathrm{d}\Gamma_{\mathrm{TPE}}}{\mathrm{d}\omega} = \frac{2}{3\pi c^4}\alpha^2\omega^3(\omega_0-\omega)^3 F_{\mathrm{p}}(\omega)F_{\mathrm{p}}(\omega_0-\omega)|T_{zz}|^2. \tag{73}$$

Comparing this to the free-space two-photon differential decay rate for an s → s transition (see Ref. 41), one finally finds that the enhancement of the two-photon emission spectrum is given by

$$\frac{\mathrm{d}\Gamma_{\mathrm{TPE}}/\mathrm{d}\omega}{\mathrm{d}\Gamma^0_{\mathrm{TPE}}/\mathrm{d}\omega} = \frac{1}{2}F_{\mathrm{p}}(\omega)F_{\mathrm{p}}(\omega_0-\omega). \tag{74}$$

SUPPLEMENTARY NOTE 10. ASSUMPTIONS AND LIMITATIONS OF THE THEORETICAL FRAMEWORK

Framework assumptions

The assumptions underlying the d-parameter framework are (see also Ref. 14):

Dipolar nature: The d-parameters emerge from an interface-centered multipole expansion of the quantum mechanical charge and current density. The monopole term gives the classical framework; the dipole term produces $d_\perp$ and $d_\parallel$; the general nth order multipole term is of order $\sim (k_{\mathrm{eff}}x)^n$, with x a length scale (e.g., $d_\perp$ and $d_\parallel$ at $n = 1$) and k_{eff} an effective modal wavevector (e.g., for a planar interface, the plasmon momentum k; for the dipole resonance of a sphere, the inverse radius R^{-1}). Truncation at the dipole term thus produces a leading-order formalism, i.e., we require that $\{|kd_{\perp,\parallel}|, |R^{-1}d_{\perp,\parallel}|\} \ll 1$. Since $|d_{\perp,\parallel}|$ is Ångström-scale, this is generally very well-satisfied.

Local curvature: The use of d-parameters—whose properties derive from *planar* interfaces—at curved interfaces implies an assumption of "local flatness". For a sphere, this is equivalent to requiring $R \gg |d_{\perp,\parallel}|$, i.e., imposes no additional restrictions relative to the dipole-expansion itself.

d-parameter nonlocality: The d-parameters are themselves k-dependent, i.e., nonlocal. This nonlocality only produces corrections of order $O(k_{\mathrm{eff}}^3)$, cf. the overall $O(k_{\mathrm{eff}}d_{\perp,\parallel})$ scaling of d-parameter terms and the expansion $d_{\perp,\parallel}(k) = d_{\perp,\parallel}(0) + \frac{1}{2}d''_{\perp,\parallel}(0)k_{\mathrm{eff}}^2 + \ldots]$. The impact of intrinsic d-parameter nonlocality is consequently comparable to the omitted quadrupole and octupole surface terms (i.e., negligible).

Surface-centric: Since the d-parameters are *surface*-response quantities they cannot describe finite-volume effects such as the so-called quantum-size effect [42; 43] (fragmentation of the bulk electronic band structure into discrete levels) nor quantized volume plasmons (the splitting of the bulk plasmon dispersion $\omega_{\mathrm{p}}(k)$ into a set of discrete "levels").[11]

The assumptions underlying our treatment of the emitter are:

Point-emitter: We use macroscopic QED to couple the states of the emitter and the electromagnetic modes of the plasmonic object (Supplementary Note SUPPLEMENTARY NOTE 8). While macroscopic

[11] Though irrelevant in the single-interface systems that we consider, the d-parameter framework of course also does not account for tunneling [44] which is intrinsically a two-interface effect.

QED does not require a point-emitter approximation, we have adopted one in our calculations.[12] As a result, emitter-size effects (see e.g., Ref. 34) are neglected. While these effects are interesting—and can be substantial in large emitters such as quantum dots or molecules—we have omitted them to unambiguously identify the impact of mesoscopic corrections from the metallic surface-response alone.

Surface hybridization: We neglect wave-function overlap between the electronic states of the emitter and metallic surface; the inclusion of which would materialize as a (complex) self-energy renormalization of the emitter's energy levels. Such a renormalization is negligible for the separations considered in our present work, where wave-function overlap is always vanishingly small. Of course, if the emitter is adsorbed to the metal surface—i.e., resides within its spill-out region—this is likely a sizable effect. Similarly, we ignore image-charge effects on the emitter's electronic orbitals. In sum, we treat the emitter's intrinsic electronic structure as independent from the metallic surface.

Note that we do not generally constrain our considerations to two-level emitters; e.g., our consideration of multipolar transitions and two-photon emission feature the hydrogen levels. Naturally, all considered processes *could* be mapped to an equivalent two-level system (except two-photon emission which includes virtual transitions between *all* levels).

Regime of validity

The sum of the above-noted assumptions translates to the broad restrictions $\{|k_{\text{eff}} d_{\perp,\parallel}|, |k_{\text{eff}} l_{\text{orb}}|\} \ll 1$, with l_{orb} denoting a characteristic size of the emitter orbitals. The effective wavevector k_{eff} of a given transition depends on the transition type and the emitter–surface separation h: e.g., for an nth order multipole transition $k_{\text{eff}} \sim n/h$ [corresponding to the dominant wavevector in Eq. (61); equivalently, the maximum of the geometric factor $q^{2n}\mathrm{e}^{-2qh}$]. As a result of this scaling with n, high-order multipole transitions will enter a regime beyond the framework's validity sooner than the dipole transition. Figure 4b,f of the main paper illustrates this phenomenom by highlighting regimes of *in*validity with dotted lines.

SUPPLEMENTARY REFERENCES

* pa@mci.sdu.dk
† tchr@mit.edu
‡ asger@mailaps.org
[1] P. A. D. Gonçalves and N. M. R. Peres, *An Introduction to Graphene Plasmonics*, 1st ed. (World Scientific, Singapore, 2016).
[2] A. A. Maradudin, W. L. Barnes, and J. R. Sambles, *Modern Plasmonics* (Elsevier, 2014).
[3] T. Christensen, W. Yan, A.-P. Jauho, M. Soljačić, and N. A. Mortensen, Phys. Rev. Lett. **118**, 157402 (2017).
[4] A. Liebsch, *Electronic excitations at metal surfaces* (Springer, New York, 1997).
[5] E. J. C. Dias, D. A. Iranzo, P. A. D. Gonçalves, Y. Hajati, Y. V. Bludov, A.-P. Jauho, N. A. Mortensen, F. H. L. Koppens, and N. M. R. Peres, Phys. Rev. B **97**, 245405 (2018).
[6] S. I. Bozhevolnyi, L. Martin-Moreno, and F. Garcia-Vidal, *Quantum Plasmonics* (Springer, 2017).
[7] M. A. Marques, C. A. Ullrich, F. Nogueira, A. Rubio, K. Burke, and E. K. U. Gross, *Time-Dependent Density Functional Theory*, Lecture Notes in Physics (Springer, New York, 2006).
[8] A. Varas, P. García-González, J. Feist, F. García-Vidal, and A. Rubio, Nanophotonics **5**, 409 (2016).
[9] P. J. Feibelman, Prog. Surf. Sci. **12**, 287 (1982).
[10] W. Yan, M. Wubs, and N. Asger Mortensen, Phys. Rev. Lett. **115**, 137403 (2015).
[11] B. N. J. Persson and P. Apell, Phys. Rev. B **27**, 6058 (1983).
[12] N. W. Ashcroft and N. D. Mermin, *Solid State Physics* (Harcourt College Publishers, New York, 1976).

[12] Obviously, for the $n > 1$ multipolar transitions, we cannot (and do not) ignore the spatial extent of the transition orbitals. Even there, however, we make an approximation that is essentially analogous to the point-emitter approximation familiar from dipole-transitions: the nth derivative of the field is assumed constant across the emitter's extent [see Eq. (60)]. For the hydrogen-like orbitals that we consider, this is an exceedingly good approximation [33].

[13] D. Jin, Q. Hu, D. Neuhauser, F. von Cube, Y. Yang, R. Sachan, T. S. Luk, D. C. Bell, and N. X. Fang, Phys. Rev. Lett. **115**, 193901 (2015).
[14] Y. Yang, Z. Di, W. Yan, A. Agarwal, M. Zheng, J. D. Joannopoulos, P. Lalanne, T. Christensen, K. K. Berggren, and M. Soljačić, arXiv:1901.03988 (2019).
[15] J. D. Jackson, *Classical Electrodynamics*, 3rd ed. (Wiley, New York, 1998).
[16] P. B. Johnson and R. W. Christy, Phys. Rev. B **6**, 4370 (1972).
[17] T. V. Teperik, P. Nordlander, J. Aizpurua, and A. G. Borisov, Phys. Rev. Lett. **110**, 263901 (2013).
[18] T. Reiners, C. Ellert, M. Schmidt, and H. Haberland, Phys. Rev. Lett. **74**, 1558 (1995).
[19] S. Mandal, J. Wang, R. E. Winans, L. Jensen, and A. Sen, J. Phys. Chem. C **117**, 6741 (2013).
[20] A. Liebsch, Phys. Rev. B **48**, 11317 (1993).
[21] S. Raza, S. Kadkhodazadeh, T. Christensen, M. Di Vece, M. Wubs, N. A. Mortensen, and N. Stenger, Nat. Commun. **6**, 8788 (2015).
[22] L. Genzel, T. P. Martin, and U. Kreibig, Z. Phys. B: Condens. Matter **21**, 339 (1975).
[23] J. A. Scholl, A. L. Koh, and J. A. Dionne, Nature **483**, 421 (2012).
[24] A. Campos, N. Troc, E. Cottancin, M. Pellarin, H.-C. Weissker, J. Lermé, M. Kociakand, and M. Hillenkamp, Nat. Phys. (2018).
[25] C. F. Bohren and D. R. Huffman, *Absorption and scattering of light by small particles* (Wiley, New York, 1983).
[26] J. A. Stratton, *Electromagnetic Theory* (McGraw-Hill, New York, 1941).
[27] L. Novotny and B. Hecht, *Principles of Nano-Optics*, 2nd ed. (Cambridge University Press, 2012).
[28] R. Carminati, A. Cazé, D. Cao, F. Peragut, V. Krachmalnicoff, R. Pierrat, and Y. D. Wilde, Surf. Sci. Rep. **70**, 1 (2015).
[29] H. Chew, J. Chem. Phys. **87**, 1355 (1987).
[30] T. Christensen, W. Yan, S. Raza, A.-P. Jauho, N. A. Mortensen, and M. Wubs, ACS Nano **8**, 1745 (2014).
[31] J. Peřina, ed., *Coherence and Statistics of Photons and Atoms* (Wiley, 2001).
[32] S. Scheel and S. Buhmann, Acta Phys. Slovaca **58**, 675 (2008).
[33] N. Rivera, I. Kaminer, B. Zhen, J. D. Joannopoulos, and M. Soljačić, Science **353**, 263 (2016).
[34] C. Van Vlack, P. T. Kristensen, and S. Hughes, Phys. Rev. B **85**, 075303 (2012).
[35] B.N.J. Persson and P. Apell, Phys. Rev. B **27**, 6058 (1983).
[36] B.N.J. Persson and E. Zaremba, Phys. Rev. B **40**, 5669 (1984).
[37] P. K. Mogensen and A. N. Riseth, JOSS **3**, 615 (2018).
[38] H. T. Dung, L. Knöll, and D.-G. Welsch, Phys. Rev. A **65**, 043813 (2002).
[39] M. Wubs and W. L. Vos, New J. Phys. **18**, 053037 (2016).
[40] J. Ren, T. Wu, B. Yang, and X. Zhang, Phys. Rev. B **94**, 125416 (2016).
[41] N. Rivera, G. Rosolen, J. D. Joannopoulos, I. Kaminer, and M. Soljačić, Proc. Natl. Acad. Sci. U. S. A. **114**, 13607 (2017).
[42] W.P. Halperin, Rev. Mod. Phys. **58**, 533 (1986).
[43] R. C. Monreal, T. J. Antosiewicz, and S. P. Apell, New J. Phys. **15**, 083044 (2013).
[44] R. Esteban, A.G. Borisov, P. Nordlander, and J. Aizpurua, Nat. Commun. **3**, 825 (2012).
